# Exosomes in Precision Oncology and Beyond: From Bench to Bedside in Diagnostics and Therapeutics

**DOI:** 10.3390/cancers17060940

**Published:** 2025-03-10

**Authors:** Emile Youssef, Dannelle Palmer, Brandon Fletcher, Renee Vaughn

**Affiliations:** Kapadi, Inc., Raleigh, NC 27615, USA

**Keywords:** exosomes, tumor microenvironment (TME), tumor-derived exosomes (TDEs), extracellular vesicles (EVs), cancer diagnostics, therapeutic resistance, liquid biopsies, biomarkers, immune modulation, drug delivery, precision medicine

## Abstract

Exosomes have emerged as pivotal players in precision oncology, offering innovative solutions to longstanding challenges such as metastasis, therapeutic resistance, and immune evasion. These nanoscale extracellular vesicles facilitate intercellular communication by transferring bioactive molecules that mirror the biological state of their parent cells, positioning them as transformative tools for cancer diagnostics and therapeutics. Recent advancements in exosome engineering, artificial intelligence (AI)-driven analytics, and isolation technologies are breaking barriers in scalability, reproducibility, and clinical application. Bioengineered exosomes are being leveraged for CRISPR-Cas9 delivery, while AI models are enhancing biomarker discovery and liquid biopsy accuracy. Despite these advancements, key obstacles such as heterogeneity in exosome populations and the lack of standardized isolation protocols persist. This review synthesizes pioneering research on exosome biology, molecular engineering, and clinical translation, emphasizing their dual roles as both mediators of tumor progression and tools for intervention. It also explores emerging areas, including microbiome–exosome interactions and the integration of machine learning in exosome-based precision medicine. By bridging innovation with translational strategies, this work charts a forward-looking path for integrating exosomes into next-generation cancer care, setting it apart as a comprehensive guide to overcoming clinical and technological hurdles in this rapidly evolving field.

## Simple Summary

Exosomes are tiny particles released by cells that play a key role in communication within the body. Scientists have discovered that these particles carry important biological messages, making them valuable tools for detecting and treating diseases, including cancer. Exosomes influence how tumors grow, resist treatments, and escape the immune system, but their role extends beyond cancer to other medical conditions. Recent advances allow researchers to engineer exosomes to deliver drugs more effectively, improve disease detection through blood-based tests, and even modify genes using advanced genetic tools. Artificial intelligence is also transforming exosome research by analyzing large datasets to identify disease patterns, improve detection accuracy, and predict treatment responses. Despite these advancements, challenges remain, including ensuring the purity and consistency of exosomes for clinical use. By combining artificial intelligence with exosome-based innovations, scientists aim to develop more precise and personalized approaches to medicine, offering new hope for earlier diagnosis and more effective treatments across multiple diseases.

## 1. Introduction

Extracellular vesicles (EVs) are lipid bilayer-delimited particles naturally released from cells, playing a pivotal role in intercellular communication and the regulation of various physiological and pathological processes [1]. Exosomes, nanoscale EVs originating from the endosomal system, and microvesicles, larger EVs budding directly from the plasma membrane, represent distinct subtypes of EVs distinguished primarily by their size and biogenesis pathways [2]. Distinguishing between these subtypes is challenging due to overlapping size ranges, shared lipid bilayer structures, and a lack of definitive markers. The International Society for Extracellular Vesicles (ISEV) recommends using “extracellular vesicles” (EVs) as a general term when precise distinctions are not feasible [3,4,5,6].

Once considered mere byproducts of cellular waste, exosomes are now recognized as active participants in both physiological regulation and disease progression [7]. These EVs, including exosomes secreted by virtually all cell types, function as molecular couriers by encapsulating diverse biomolecules—such as proteins, lipids, and nucleic acids—that mirror the biological state of their parent cells. By mediating intercellular communication, exosomes influence critical processes such as gene regulation, immune responses, angiogenesis, apoptosis, and metabolic reprogramming. Exosomes play a crucial role in wound healing, host–microbiome interactions, tumor progression, metastasis, and maintaining homeostasis, while dynamically responding to pathological states [8,9]. This multifunctionality highlights their significance in both health and disease, positioning exosomes as pivotal mediators with wide-ranging implications in oncology and therapeutic innovation biology [10].

Tumor-derived exosomes (TDEs), a key subtype of EVs, are instrumental in reshaping the tumor microenvironment (TME) by facilitating critical processes such as immune suppression, angiogenesis, and metabolic reprogramming [2,7]. Moreover, they contribute to tumor metastasis and therapeutic resistance, reflecting their multifaceted impact on cancer progression [7]. These vesicles also carry specific molecular signatures that mirror the genetic and phenotypic traits of their originating tumor cells, highlighting their potential as noninvasive biomarkers for liquid biopsies [7]. Through such applications, exosomes offer unprecedented opportunities for early cancer detection, real-time disease monitoring, and therapy customization [10]. While their promise is immense, the clinical translation of exosome-based diagnostics and therapies faces significant challenges [11].

Key challenges in the field include standardizing isolation techniques, addressing the heterogeneity of EVs, and achieving scalability for large-scale production [12]. Advancements in nanotechnology, bioengineering, and high-throughput analytical methods are steadily overcoming these barriers, positioning EVs and exosomes as promising contributors to precision medicine [13].

Progress in EV research relies on fostering interdisciplinary collaboration among scientists, clinicians, bioengineers, and industry leaders. Such synergy is vital for addressing current challenges and unlocking the full potential of EVs in diagnostics and beyond. This review bridges exosome biology and clinical applications, synthesizing recent findings on their roles in cancer diagnostics, immune modulation, and innovative bioengineering approaches. By highlighting advancements in isolation techniques, AI-driven analytics, and bioengineered exosomes, it provides a roadmap for overcoming existing limitations [13]. Ultimately, these developments lay the foundation for integrating exosome-based innovations into routine clinical practice, paving the way for precision oncology and broader medical applications.

## 2. Exosome Biogenesis and Molecular Composition

**Figure 1:**
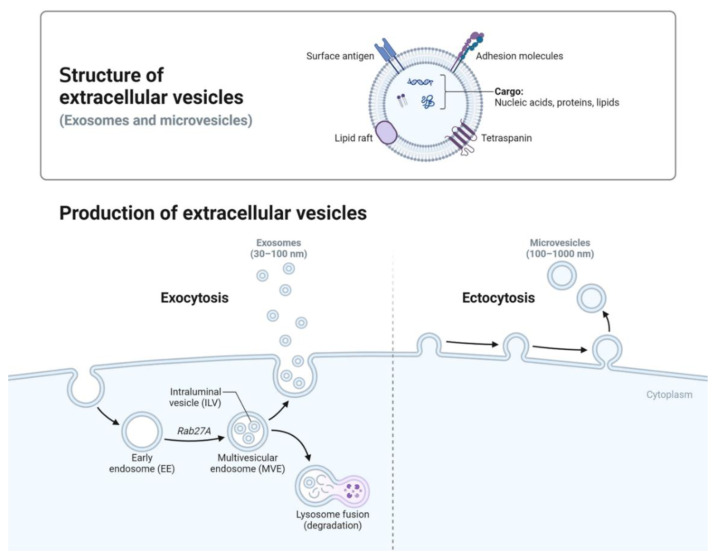
Biogenesis and Characteristics of Extracellular Vesicles. This schematic illustrates the biogenesis pathways of extracellular vesicles (EVs), including exosome formation through the endosomal system and microvesicle generation via outward budding from the plasma membrane. Exosomes (30–100 nm) originate from multivesicular bodies (MVBs) and are released into the extracellular space, while microvesicles (100–1000 nm) bud directly from the plasma membrane. Both EV types carry diverse bio-molecular cargo, reflective of their cellular origin. The distinct mechanisms of biogenesis underpin the therapeutic potential of EVs, enabling the selective packaging and targeted delivery of bioactive molecules for applications in cancer therapy, gene editing, and immunomodulation. The figure was created using www.BioRender.com [26].

Exosomes are nanoscale vesicles with intricate biogenesis pathways and diverse molecular compositions, reflecting the physiological or pathological states of their originating cells. Figure 1 illustrates the formation pathways and structural features of two primary EV subtypes: exosomes and microvesicles. Exosomes (30–100 nm) originate from the endosomal system, where the inward budding of endosomal membranes forms intraluminal vesicles within MVBs [14]. In MVBs, intraluminal vesicles (ILVs) form via two pathways: endosomal sorting complex required for transport (ESCRT)-dependent and ESCRT-independent. ESCRT drives inward budding to encapsulate cytoplasmic components, while lipids and tetraspanins support ESCRT-independent formation [15,16,17,18]. Upon fusion of multivesicular bodies (MVBs) with the plasma membrane [14], these vesicles are released as exosomes into the extracellular space Unlike other EVs, exosomes are primarily formed through the endosomal pathway, contributing to their distinct composition enriched with tetraspanins (e.g., CD9, CD63, CD81) and signaling molecules [19,20]. In contrast, microvesicles (100–1000 nm) are generated through direct outward budding of the plasma membrane [14]. Advanced analytical techniques are essential for dissecting exosome subpopulations and distinguishing them from other EV types, such as microvesicles and apoptotic bodies [21].

EVs employ various mechanisms to enter target cells, including clathrin-mediated endocytosis, caveolin-dependent pathways, macropinocytosis, and direct membrane fusion [22,23]. Exosomes, in particular, demonstrate remarkable specificity in cargo delivery, driven by their surface markers and lipid composition, which enable selective targeting and uptake by recipient cells [22,24,25]. In contrast, microvesicles carry more heterogeneous cargo and exhibit less selectivity in their interactions with target cells.

## 3. Molecular Complexity and Emerging Roles of Extracellular Vesicles in Oncology

Exosomes are nanoscale vesicles with diverse molecular compositions that reflect the physiological or pathological states of their cells of origin. Recent studies have identified thousands of proteins, lipids, mRNAs, and microRNA (miRNAs) within exosomes, underscoring their potential as versatile therapeutic vehicles [27,28,29]. This complexity mirrors the condition of their parent cells, making exosomes valuable tools for understanding tumor behavior and guiding treatment strategies [22,28].

Exosomes share functional similarities with viral vectors in delivering nucleic acids and proteins to recipient cells. However, exosomes are less immunogenic and do not pose risks such as insertional mutagenesis, which makes them safer alternatives for gene therapy [30,31]. This highlights their potential for overcoming limitations associated with traditional delivery systems while offering improved safety and efficacy [19,20,31].

TDEs play a critical role in cancer progression by transporting oncogenic drivers, pro-angiogenic factors, and immune modulators. These vesicles are enriched with nucleic acids, including miRNAs and circRNAs, that regulate gene expression and contribute to therapeutic resistance [28,29,32,33,34,35,36]. Specific nucleic acids within TDEs also serve as stable biomarkers and potential therapeutic targets [28,29,32,33,34,35]. Moreover, TDEs facilitate pre-metastatic niche formation by delivering integrins and other proteins that promote organ-specific metastasis [17,36,37,38].

Recent advancements have enabled researchers to harness exosomes for targeted therapies. Their natural affinity for immune cells is being leveraged to modulate immune responses, while their ability to encapsulate bioactive molecules is being utilized for therapeutic delivery. For example, exosomes carrying anti-tumor miRNAs or small interfering RNAs (siRNAs) can suppress oncogenic pathways, and engineered exosomes are being developed to present tumor antigens for enhanced T-cell activation [39,40].

To overcome challenges such as exosome heterogeneity and scalability, researchers are refining techniques for isolating and functionalizing EV subtypes. Artificial exosomes are being bioengineered to deliver precise therapeutic cargo while addressing limitations in reproducibility and production scale [19,41,42,43]. These innovations pave the way for integrating exosome-based technologies into clinical applications, particularly in cancer diagnosis and therapy treatment [44].

In summary, the molecular complexity of exosomes underscores their dual roles as both mediators of tumor progression and tools for intervention. By elucidating the mechanisms of EV-mediated communication, researchers are developing more effective therapies and diagnostics. This focus bridges the gap between molecular insights and clinical innovation, advancing the field of precision oncology.

## 4. Dual Roles of Exosomes in Oncology

Exosomes, a subset of extracellular vesicles, play dual roles in cancer biology, acting as both promoters of tumor progression and mediators of anti-tumor effects. Their functional outcomes depend on their cellular origin, molecular cargo, and the context of their interactions with recipient cells. This complexity positions them as both targets and tools in oncology.

Exosomes from healthy or engineered cells have shown significant tumor-suppressive potential. These exosomes deliver anti-tumor molecules, such as tumor-suppressive miRNAs (e.g., miR-146a) or siRNAs targeting oncogenes like KRAS, effectively downregulating tumor-promoting pathways [39]. Engineered exosomes presenting tumor antigens can enhance T-cell activation and improve the effectiveness of Checkpoint Inhibitors (CPIs), thereby stimulating robust anti-tumor immune responses [45]. Additionally, exosomes loaded with chemotherapeutic agents, such as doxorubicin or paclitaxel, enable targeted drug delivery to tumor sites, minimizing systemic toxicity and improving therapeutic outcomes [46]. Exosomes derived from mesenchymal stem cells (MSCs) are particularly notable for their ability to counteract immunosuppressive signals within the TME and promote anti-tumor immunity [47]. Leveraging tumor-suppressive exosomes through bioengineering enables the precise delivery of genetic material, including Clustered Regularly Interspaced Short Palindromic Repeats and CRISPR-associated protein 9 (CRISPR-Cas9) components, for targeted oncogene editing.

Table 1 highlights the diverse mechanisms by which engineered exosomes influence immune responses, showcasing their roles in immune modulation. Each mechanism includes a description of the exosomal role in facilitating immune activity and provides a specific example demonstrating its application. From enhancing T-cell and NK cell activity to counteracting regulatory T cells (Tregs) and synergizing with immune CPIs, the table emphasizes the potential of exosomes in advancing immunotherapy strategies.

In contrast, TDEs actively drive tumor progression through their dynamic modulation of the TME. They promote metastasis by delivering integrins to recipient cells, creating pre-metastatic niches in distant organs [17]. TDEs also carry oncogenic drivers and pro-angiogenic factors that support tumor growth and vascular remodeling [45] and contribute to immune evasion and therapeutic resistance [48]. Exosomes exhibit a complex and transformative potential in oncology, acting as both oncogenic drivers and tumor suppressors. By elucidating the mechanisms behind these dual roles, researchers can design more effective therapies and refine diagnostic approaches. While exosomes enhance immune activation through antigen presentation and can be engineered to deliver siRNAs targeting oncogenes such as KRAS and MYC, they also contribute to reprogramming the tumor microenvironment to counteract immunosuppression. However, exosomes can also facilitate tumor progression by promoting metastasis through integrin-mediated pathways and carrying oncogenic drivers such as KRAS, VEGF, and PD-L1. These TDEs suppress T-cell activity, thereby contributing to immune evasion. Addressing these contrasting roles is essential for advancing precision medicine. The therapeutic potential of exosomes is counterbalanced by challenges related to the efficient loading and delivery of therapeutic cargo, as well as their unintended role in promoting therapy resistance. Strategies such as engineering exosomes for precision drug delivery and targeting TDE biogenesis are actively being explored to mitigate these oncogenic effects while leveraging their beneficial roles in therapy. This nuanced focus bridges the gap between molecular insights and clinical innovation, driving progress in precision medicine.

These insights emphasize the versatility of exosomes as a platform for innovative therapies targeting the TME and immune system.

## 5. Advances in Exosome Isolation Technologies and Their Role in Oncology

Innovative approaches to EVs and exosome isolation are addressing critical challenges that have long hindered their clinical translation. Traditional isolation techniques, including ultracentrifugation, size-exclusion chromatography (SEC), and immunoaffinity capture, remain foundational for exosome research. However, each method faces notable limitations. For example, ultracentrifugation, despite its accessibility, often requires long processing times and may co-isolate contaminants like protein aggregates, affecting purity [49,50]. SEC offers gentler handling and better exosome integrity but struggles to separate exosomes from similarly sized particles, limiting its precision [51]. Immunoaffinity capture provides high specificity by targeting surface markers such as CD63 and CD81 but is constrained by high costs and inefficiency for large-scale applications [52].

To overcome these challenges, emerging technologies such as microfluidic platforms and acoustic-based isolation methods are transforming the field. Microfluidic devices, for instance, integrate size-based filtration, density separation, and immunoaffinity techniques on a single chip, enabling rapid, high-throughput isolation from minimal sample volumes [53]. Similarly, acoustic-based methods utilize ultrasonic waves to achieve size and density separation while maintaining exosome integrity, with recovery rates exceeding 85% for particles in the 100 nm range [54]. Hybrid approaches, like combining ultracentrifugation with SEC or immunoaffinity capture, further enhance yield and reproducibility, addressing clinical-scale production needs [52,54].

Building on these foundations, newer methods such as tangential flow filtration (TFF) and size-exclusion fast performance liquid chromatography (SE-FPLC) offer high scalability and throughput, addressing the limitations of traditional approaches [55,56]. By integrating these innovations with standardized protocols, the scalability and clinical applicability of exosome-based technologies are steadily advancing. These advancements are complemented by sophisticated analytical tools such as quantitative reverse transcription-polymerase chain reaction (qRT-PCR), droplet digital polymerase chain reaction (ddPCR), and Machine Learning (ML) algorithms, which significantly enhance the sensitivity and reproducibility of EVs analysis [57]. Additionally, in vivo flow cytometry (IVFC) enables real-time monitoring of small EVs, providing valuable insights into their pharmacokinetics, biodistribution, and cellular targeting—key factors for optimizing therapeutic and diagnostic strategies [58]. The Vesiclepedia database further supports these innovations by curating bio-molecular datasets, including RNA, proteins, and lipids, and metabolites, to accelerate biomarker discovery and characterization [59]. Integrated with ML models trained on exosomal profiles, these tools facilitate precise cancer subtype identification and significantly advance EV-based precision medicine [60].

**Figure 2:**
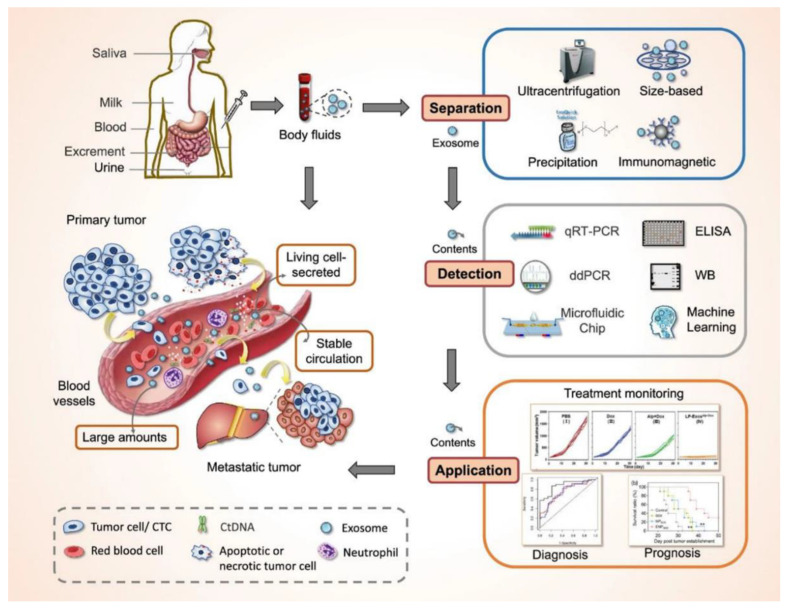
Overview of exosomes as novel biomarkers for liquid biopsy. Exosomes, secreted by living cells, are abundantly present in various body fluids, including blood, saliva, urine, and milk, enabling their stable circulation in the bloodstream. The figure outlines the workflow for exosome-based liquid biopsies, starting with separation techniques such as ultracentrifugation, size-based filtration, precipitation, and immunomagnetic methods. Following isolation, exosomal content is analyzed using advanced detection tools, including qRT-PCR, ddPCR, ELISA, Western blot (WB), microfluidic chips, and machine learning (ML) approaches. These methods facilitate the use of exosomes for treatment monitoring, diagnosis, and prognosis of cancer. This comprehensive approach indicates the transformative potential of exosomes as a target for minimally invasive cancer diagnostics and personalized therapy. Figure 2 was adapted from Yu et al., 2022 [57].

Figure 2 illustrates these techniques and their transformative impact on oncology care. Of note, milk-derived and plant-derived EVs are highlighted as potential candidates for oral drug delivery due to their biocompatibility and stability in gastrointestinal fluids. Techniques like layer-by-layer coating have enhanced their efficacy [61]. Plant-derived EVs are rich in lipids like digalactosyldiacylglycerol, which contribute to their unique therapeutic effects [62]. Plant-derived and milk-derived EVs are distinguished by their low cost, scalability, and potential for use in noninvasive therapies.

Looking forward, efforts are focused on developing automated, high-throughput systems to reduce variability and streamline workflows. Advances in bioreactor systems and artificial exosome production hold promise for meeting the demand for clinical-grade exosomes, addressing the challenge of scalability [63]. By combining these technological advancements with universally accepted protocols, exosome isolation is poised to become a reliable cornerstone of precision oncology, enabling transformative advancements in cancer diagnostics and therapeutics.

## 6. Exosome Heterogeneity and Standardization: Challenges and Advance

Exosome heterogeneity remains a critical challenge in translational and clinical research, impacting reproducibility, biomarker validation, and therapeutic development. Variability in size, composition, and function—arising from different cellular origins, biogenesis pathways, and disease states—complicates the establishment of universal diagnostic and therapeutic protocols. The presence of diverse exosomal subpopulations, even within a single biofluid sample, has been demonstrated through single-particle imaging and proteomic profiling [64]. TDEs, for instance, carry distinct oncogenic proteins, miRNAs, and metabolic factors, with variations observed across cancer types and disease progression stages [65]. Hypoxia-induced exosomes from aggressive tumors are enriched with miR-210 and Hypoxia-Inducible Factor (HIF)-1α, which promote angiogenesis and immune suppression [66], while therapy-resistant exosomes contain altered lipid compositions and drug-efflux pumps that contribute to chemoresistance [67]. High-dimensional flow cytometry, single-vesicle imaging, and AI-driven proteomic clustering are emerging as promising approaches to classify exosomal subpopulations based on molecular signatures rather than traditional size- or density-based methods [68,69,70].

Efforts to standardize exosome research have resulted in the implementation of global initiatives. The Minimal Information for Studies of Extracellular Vesicles (MISEV) guidelines, established by the International Society for Extracellular Vesicles (ISEV), set essential criteria for exosome characterization, including surface markers (CD9, CD63, CD81), RNA profiling, and rigorous particle quantification [71]. Additionally, EV-TRACK, a centralized database, enables researchers to submit exosome characterization data to improve reproducibility [72]. Advances in isolation techniques, such as TFF, asymmetric flow field-flow fractionation (AF4), and label-free nanoparticle tracking, are improving exosome purity and yield [73]. Hybrid techniques—such as combining ultracentrifugation with immunoaffinity capture—have also enhanced reproducibility in clinical exosome preparations [12,74]. Good Manufacturing Practices (GMP)-compliant production pipelines are now critical for clinical translation, with biopharmaceutical companies integrating real-time quality control measures such as nanoparticle tracking analysis and flow cytometry validation to ensure batch consistency [75].

Artificial intelligence is playing an increasingly vital role in standardizing exosome research. ML and deep learning (ML) models can classify exosomes based on multi-omics data, with AI-assisted image processing improving the precision of exosome quantification [76]. AI-powered single-vesicle proteomics, for example, has demonstrated an ability to identify tumor-specific exosomal signatures with high accuracy, enhancing the potential for personalized cancer diagnostics [60]. These computational tools allow researchers to predict functional differences in exosomal cargo more effectively and reduce operator-dependent variability in exosome characterization.

Despite these advancements, standardization remains challenging due to batch-to-batch variability, storage and stability issues, functional inconsistencies, and regulatory uncertainty. Exosomes derived from natural sources, such as mesenchymal stem cells and tumors, exhibit compositional differences that can affect therapeutic efficacy, necessitating strict GMP protocols and real-time quality control assays [77,78]. Unlike synthetic nanoparticles, exosomes require specialized cryopreservation methods to prevent aggregation or degradation, with lyophilization and advanced stabilization agents currently being explored to enhance shelf-life [79]. The biological effects of exosomes depend not only on their molecular cargo but also on uptake efficiency and biodistribution, making functional standardization difficult. Live-cell imaging and organ-on-chip models are being employed to better understand exosome biodistribution and uptake kinetics [80,81]. Additionally, the lack of clear regulatory frameworks for exosome-based therapeutics has created challenges in clinical trial design and approval, requiring the establishment of well-defined potency assays and mechanism-of-action studies to facilitate regulatory acceptance [82,83,84].

Significant progress has been made in overcoming the complexities of exosome heterogeneity, with AI-driven analytics, improved isolation protocols, and GMP-compliant production pipelines addressing key challenges. Successful case studies from biopharmaceutical companies, global research initiatives, and high-throughput exosome datasets are paving the way for reproducible, high-quality exosome applications in cancer diagnostics and therapy. However, continued interdisciplinary collaboration among scientists, regulatory agencies, and industry partners will be required to fully integrate exosome-based technologies into precision medicine.

## 7. Extracellular Vesicles and Exosomes in Cancer Diagnostics

EVs, including exosomes, have become integral to the diagnostic landscape in oncology, offering a critical link between molecular biology and clinical application. Their stability and noninvasive nature make exosomes an ideal platform for biomarker identification, real-time disease monitoring, and the development of personalized diagnostic strategies. This section focuses on their transformative role in cancer diagnostics, emphasizing their advantages, key biomarkers, and innovative applications in improving detection and patient stratification.

### 7.1. Advantages of Exosome-Based Liquid Biopsies

Liquid biopsies utilizing EVs, especially exosomes, offer notable advantages over conventional diagnostic approaches [85,86]. In recent years, exosome-based diagnostics have emerged as a transformative alternative to traditional tissue biopsies, particularly in oncology [87]. Despite their promise, challenges persist in standardizing and scaling exosome isolation, as well as addressing heterogeneity within exosomal populations [86,88]. Exosome-based liquid biopsies leverage their stability, sensitivity, and noninvasive nature to enable real-time monitoring of tumor dynamics and molecular heterogeneity. These properties make exosomes a transformative platform for cancer diagnostics, offering advantages over traditional tissue biopsies. Recent advancements, including AI-driven analytics and high-throughput detection techniques, further enhance diagnostic accuracy and clinical applicability [89].

### 7.2. Key Biomarkers in EVs- and Exosome-Based Diagnostics

Recent innovations in spatial EV analysis, such as using cellulose nanofiber (CNF) sheets, have demonstrated the ability to capture EVs from trace amounts of biofluids and identify location-specific miRNA profiles [90]. These profiles, linked to cancer progression and the heterogeneity of the TME, provide valuable insights for understanding tumor biology, aiding in staging, and informing therapy selection. EV-based diagnostics, including exosome-specific approaches, have identified a range of biomarkers with significant clinical relevance, enabling precise cancer detection and monitoring. Below are key examples of exosomal biomarkers and their roles in oncology [90]:

Glypican-1 (GPC-1): Found in serum-derived exosomes, GPC-1 demonstrates near-perfect sensitivity and specificity for early pancreatic cancer detection and is also a promising biomarker for colorectal cancer (CRC) [91].

miR-210: Highly expressed in plasma exosomes from pancreatic cancer patients, miR-210 serves as a reliable biomarker for early detection. It is associated with tumor hypoxia and metabolic reprogramming, key features of pancreatic cancer progression [92].

miR-15a-5p is overexpressed in exosomes derived from endometrial cancer patients, achieving an Area Under the Curve (AUC) of 0.813 in distinguishing early-stage (stage I) endometrial cancer patients from healthy controls [93]. When combined with serum markers CEA and CA125, the diagnostic accuracy improves significantly, with the AUC increasing to 0.899, underscoring its potential to enhance diagnostic protocols [75]. Beyond its diagnostic utility, miR-15a-5p correlates with critical clinical features of endometrial cancer, such as muscular infiltration depth and tumor aggressiveness. Furthermore, its levels are associated with reproductive hormones like testosterone and dehydroepiandrosterone sulfate (DHEAS), offering insights into disease mechanisms and progression [47].

miR-92a: Detected in plasma exosomes, miR-92a demonstrates significant diagnostic utility for colorectal CRC. Its overexpression differentiates CRC patients from healthy controls and provides insights into tumor aggressiveness and metastasis potential [94].

CD63-Positive Exosomes: These exosomes, enriched with miR-21 and HER2, play a critical role in breast cancer by reflecting tumor aggressiveness and resistance mechanisms. They have also been implicated in establishing pre-metastatic niches and promoting metastasis [95]. miR-21 exhibits elevated expression across various cancer types compared to normal tissue, with high levels correlating with poor patient prognosis. The oncogenic properties of miR-21 include targeting tumor suppressor genes such as PTEN, PDCD4, and TIMP3, promoting cellular invasion and metastasis [95].

Exosomal miR-1247-3p: It plays a pivotal role in cancer progression by modulating the TME. In hepatocellular carcinoma, it activates cancer-associated fibroblasts (CAFs) via the β1-integrin–NF-κB pathway, promoting lung metastasis. In bladder cancer, it drives angiogenesis by targeting FOXO1, enhancing tumor vascularization. Clinically, miR-1247-3p holds promise as a biomarker for metastasis and angiogenesis, offering the potential for noninvasive diagnostics and as a therapeutic target to disrupt metastatic and angiogenic pathways [96,97].

Annexin V-Positive Exosomes: Annexin V-positive exosomes carrying prostate-specific antigens (PSA and PSMA) provide valuable insights into aggressive prostate cancer phenotypes. These biomarkers hold the potential for early detection and disease characterization, assisting in the stratification of high-risk patients [98,99].

miR-141: Elevated in plasma-derived exosomes, miR-141 is a promising biomarker for prostate cancer detection and monitoring. Its expression levels correlate with disease progression, making it a valuable tool for stratifying patients and guiding personalized treatment strategies [100].

CD81-Positive Exosomes: These exosomes carry EGFR variants instrumental in tracking resistance to targeted therapies in non-small cell lung cancer (NSCLC), enabling timely adjustments in treatment strategies [101,102].

Exosomes Enriched with HER2: TDEs with HER2 facilitate breast cancer monitoring and treatment planning, offering insights into resistance mechanisms and therapeutic effectiveness [103].

miR-155: Enriched in plasma exosomes of breast cancer patients, miR-155 is linked to tumor progression, aggressiveness, and poor prognosis. Its oncogenic role includes targeting tumor suppressor pathways, making it a potential biomarker for tracking disease outcomes and therapeutic responses [104,105].

This dynamic reflection of tumor biology is invaluable for assessing disease progression and therapeutic responses. Moreover, the integration of bioinformatics and ML in exosome research has enabled the identification of complex biomarker signatures, advanced precision oncology and supporting the development of personalized treatment strategies. Table 2 highlights a comprehensive overview of exosomal biomarkers and their clinical utility in oncology, with applications in liquid biopsy diagnostics. It details various exosome sources, their associated biomarkers, and their diagnostic roles in cancer detection, treatment monitoring, and patient stratification. Synthetic exosomes carrying tumor DNA alterations demonstrate potential for cancer subtype stratification, emphasizing their role in precision oncology. By leveraging the molecular heterogeneity of tumors, these exosome-based biomarkers provide a transformative approach to real-time monitoring, therapeutic decision-making, and personalized cancer care.

### 7.3. Exosomal Signatures in Cancer Diagnostics and Therapy

Table 3 Summary of the diagnostic and therapeutic relevance of exosomal signatures in oncology and a number of non-oncology Indications. The table presents validated exosomal biomarkers across multiple cancer types, highlighting their specific molecular signatures, clinical implications, and supporting literature references.

Exosomal cargo serves as a highly specific and sensitive biomarker for cancer detection and prognosis. In renal cell carcinoma (RCC), for example, a panel of mRNA markers (CUL9, KMT2D, PBRM1, PREX2, and SETD2) has been identified as a highly accurate classifier, distinguishing RCC from benign renal masses with an AUC of 0.83 [127]. Similarly, in prostate cancer, exosomal CDC42, IL32, MAX, NCF2, PDGFA, and SRSF2 significantly outperform traditional PSA-based screening, demonstrating a superior AUC of 0.95 [128].

Exosomal metabolites like succinic acid and lactate have also been associated with therapy resistance in breast cancer. Patients exhibiting high levels of these metabolic markers in circulating exosomes were found to have poor responses to neoadjuvant chemotherapy (NAC) [129]. These findings underscore the potential of metabolic profiling in exosomes as a tool for predicting chemotherapy efficacy.

One of the most compelling applications of exosomal biomarkers lies in their ability to predict therapy resistance. Exosomes are known to facilitate tumor adaptation through metabolic reprogramming, affecting glycolysis and lipid metabolism pathways in breast, pancreatic, and lung cancers [130]. This metabolic shift enables cancer cells to survive and proliferate despite therapeutic interventions. For instance, in pancreatic cancer, exosomal KRAS mutations have been proposed as liquid biopsy alternatives for early detection, as they capture the molecular landscape of tumor evolution [130]. Similarly, exosomal PD-L1 levels have been strongly correlated with immune evasion in checkpoint inhibitor therapy, providing a predictive marker for anti-PD-1 therapy responsiveness [131].

In addition to their diagnostic utility, exosome-based therapeutic strategies are gaining momentum. The development of siRNA-loaded exosomes targeting oncogenic KRAS and MYC represents a multi-target precision therapy approach [132]. Exosomal miRNAs have been successfully used to distinguish aggressive tumor subtypes and track disease progression. A study on multiple sclerosis demonstrated that exosome-associated miRNAs can differentiate disease subtypes with high accuracy using integrative bioinformatics and ML approaches. Similarly, in oncology, exosomal miRNA signatures, such as miR-21 and miR-155, have been linked to tumor aggressiveness, immune evasion, and treatment resistance [133]. Additionally, advanced statistical modeling, including Random Forest analysis and hierarchical clustering, has been employed to validate exosomal miRNA biomarkers as liquid biopsy tools, demonstrating their potential to enhance early cancer detection and patient stratification. By leveraging next-generation sequencing and AI-assisted classification, exosomal biomarkers continue to evolve as powerful precision oncology tools, enabling the non-invasive monitoring of tumor progression and treatment efficacy.

These engineered exosomes can effectively silence tumor-driving genes, thereby enhancing treatment efficacy and reducing off-target effects. To consolidate the growing body of knowledge on exosomal biomarkers, Table 3 provides an organized summary of validated exosomal signatures, their respective cancer-type associations, and their clinical significance. This comprehensive summary allows researchers to quickly reference key exosomal biomarkers across different cancer types, understand their clinical implications, and explore potential therapeutic interventions. Despite significant advancements, several challenges must be addressed before exosomal biomarkers can be fully integrated into routine clinical practice [134]. Standardization of exosome isolation and analysis is essential to improve reproducibility across studies. Validation in large patient cohorts is needed to confirm their diagnostic and therapeutic efficacy. Additionally, the integration of multi-omics approaches, combining exosomal RNA, protein, and metabolic profiling, will enhance biomarker discovery and clinical applicability.

**Table 3:** Exosomal signatures in disease diagnostics and therapy.

Cancer Type/Disease	Exosomal Signature	Relevance	References
Renal Cell Carcinoma (RCC)	CUL9, KMT2D, PBRM1, PREX2, SETD2 (mRNAs)	Distinguishes RCC from benign masses with high specificity (AUC = 0.83)	[127]
Prostate Cancer	CDC42, IL32, MAX, NCF2, PDGFA, SRSF2 (mRNAs)	Improves prostate cancer detection over PSA-based screening (AUC = 0.95)	[128]
Breast Cancer (NAC resistance)	Succinic acid, Lactate (Exosomal metabolic markers)	Predicts chemotherapy resistance in patients with residual disease	[129]
Pancreatic Cancer	Exosomal KRAS mutations	Liquid biopsy alternative for early detection	[130]
Multiple Sclerosis (Neurological Disease)	miR-15b-5p, miR-342-3p, miR-432-5p	Differentiates RRMS from progressive MS subtypes	[132]
Immune Checkpoint Resistance	Exosomal PD-L1	Tumor immune evasion; Targeting strategy for checkpoint blockade therapy	[130]
Metabolic Reprogramming & Therapy Resistance	Glycolysis & Lipid metabolism pathways (Exosomal cargo)	Key driver of resistance in breast, pancreatic, and lung cancer	[133]
Combinatorial Targeting Approaches	siRNA-loaded exosomes for KRAS, MYC	Multi-target precision therapies	[131]

### 7.4. Emerging Diagnostic Applications and Future Potential

As highlighted in Table 3, plasma-derived exosomes carrying miR-15a-5p have shown promise for the early detection of endometrial cancer, while tumor-derived exosomal GPC-1 has emerged as a valuable biomarker for pancreatic cancer [135]. These findings highlight the transformative role of exosomes in minimally invasive, real-time cancer diagnostics, offering clinicians tools to detect tumors at early stages with high specificity and sensitivity [47,135].

miR-210 in plasma exosomes has demonstrated utility in the early detection of pancreatic cancer, with its association with tumor hypoxia and metabolic reprogramming providing critical insights into disease progression [92]. Similarly, miR-92a, identified in plasma-derived exosomes, serves as a significant diagnostic marker for colorectal cancer, differentiating CRC patients from healthy controls and offering insights into tumor aggressiveness and metastatic potential [94]. miR-141 is a reliable biomarker for prostate cancer, aiding in the detection and monitoring of disease progression through plasma exosomes [100]. Finally, miR-155, enriched in plasma exosomes, is linked to breast cancer progression and chemoresistance, making it a critical tool for tracking therapeutic outcomes [104,105].

Exosomal biomarkers have also proven valuable in monitoring resistance mechanisms [136]. For instance, PD-L1-positive exosomes serve as indicators of immune checkpoint activity and predictors of patient response to immunotherapy [98,137], while mutated EGFR-carrying exosomes enable the tracking of resistance to targeted therapies in NSCLC [136,138].

The accessibility of exosomes in biofluids facilitates longitudinal monitoring of tumor dynamics, treatment response, minimal residual disease, and the early detection of cancerous changes, providing a noninvasive alternative to traditional biopsies [139]. Moreover, changes in the levels of chemoresistance-associated miRNAs, such as miR-155 and miR-221, signal the emergence of therapeutic failure, enabling timely diagnostic adjustments and personalized clinical management [140]. These innovations are further bolstered by bioinformatics and ML integration, which enhance the precision of exosome-based diagnostics [141].

## 8. Extracellular Vesicles as Platforms for Cancer Therapeutics and Beyond

EVs, including exosomes, are emerging as promising platforms for cancer therapeutics and beyond, leveraging their natural ability to encapsulate and deliver bioactive cargo with high specificity and low immunogenicity. These nanoscale vesicles, secreted by various cell types, are uniquely positioned to bridge biological barriers, modulate immune responses, and enable precise targeting of diseased tissues. Recent advancements in exosome engineering and therapeutic applications have further emphasized their potential to revolutionize precision medicine by addressing challenges in drug delivery, immunotherapy, and regenerative medicine. Through their multifaceted roles, EVs are redefining therapeutic innovation across oncology and other domains.

### 8.1. Advances and Therapeutic Applications of Exosomes

EVs, particularly exosomes, have become transformative tools in cancer therapeutics due to their unique properties, including biocompatibility, low immunogenicity, the ability to cross biological barriers, and selective cargo delivery. Recent advancements in genetic engineering and surface modification have enhanced their precision as drug delivery systems. Techniques such as ligand display on exosome surfaces and chemical modifications like click chemistry enable targeted delivery of therapeutic agents or imaging moieties [142]. Engineered EVs equipped with surface proteins, such as anti-HER2 or anti-epidermal growth factor receptor (EGFR) antibodies, exhibit significantly improved tumor-targeting capabilities, positioning EVs as versatile platforms for addressing therapeutic resistance and advancing precision oncology [143].

Exosome-based therapies offer versatile therapeutic applications, leveraging their biocompatibility, low immunogenicity, and ability to cross biological barriers, making them ideal for precision medicine. As Table 4 demonstrates, among the various sources of exosomes, MSC-derived exosomes are extensively studied for their natural immunomodulatory properties and regenerative potential, enabling them to suppress inflammation, promote tissue repair, and support immune regulation in various pathological contexts. Their applications span areas such as wound healing, cartilage regeneration, and osteoporosis treatment. These exosomes offer advantages such as low immunogenicity and scalability, making them suitable for clinical translation and advanced therapeutic applications. Innovative approaches include engineering these exosomes for targeted drug delivery and preconditioning them to enhance immune responses, thereby improving outcomes in cancer immunotherapy and regenerative medicine [82,142,144].

TDEs play a crucial role in oncology, particularly in delivering siRNAs targeting oncogenic mutations like KRAS, which addresses critical drivers of cancer progression. These exosomes are essential in precision oncology, enabling gene-silencing strategies for targeted cancer treatment [103,117]. Similarly, synthetic exosomes have advanced gene therapy by enabling the precise delivery of CRISPR-Cas9 components for oncogene editing. Their integration of synthetic properties enhances scalability and targeting specificity, offering innovative solutions to address genetic mutations in cancer [103,144].

Preconditioned exosomes, derived from parent cells exposed to specific stimuli, have shown promise in amplifying immune responses and improving cancer immunotherapy for solid tumors. For example, preconditioning with interferon-gamma (IFN-γ) enhances the immunotherapeutic potential of these exosomes, enabling more effective modulation of the TME [91]. Artificial exosomes, which combine natural and synthetic components, further advance therapeutic delivery by improving targeting specificity and scalability. These hybrid vesicles represent an innovative approach to overcoming resistance mechanisms in cancer therapy [118,145,146].

Table 4 also highlights plant-derived exosomes as a scalable and ethical solution for personalized medicine. These exosomes demonstrate potential in delivering therapeutic agents such as low-density lipoprotein receptor (LDLR) mRNA for familial hypercholesterolemia, highlighting their biocompatibility and utility in drug delivery systems [61,62,117]. Similarly, milk-derived exosomes offer natural stability and compatibility, making them particularly effective for oral drug delivery. For instance, their use in delivering curcumin to treat colon tissue inflammation indicates their promise for addressing metabolic and autoimmune disorders [142,147].

**Figure 3:**
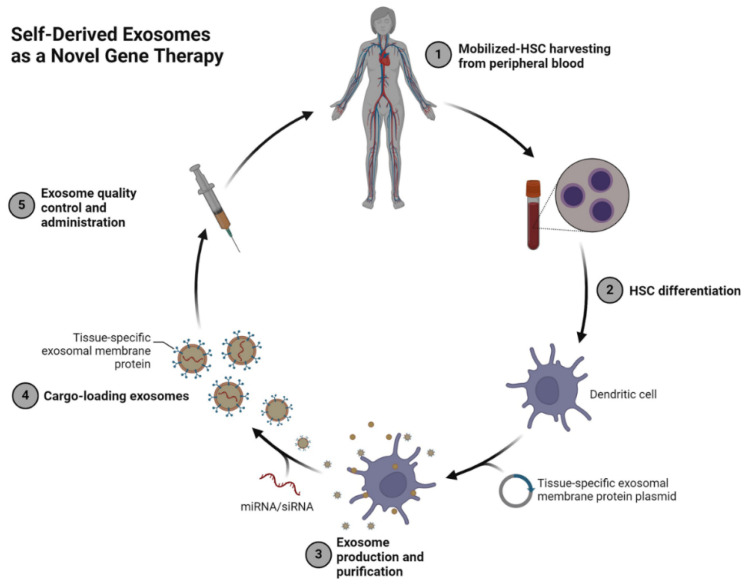
Self-Derived Exosomes as a Novel Gene Therapy Platform. This schematic represents the process of creating self-derived exosomes for therapeutic applications. Peripheral blood-derived hematopoietic stem cells (HSCs) are mobilized and harvested (Step 1) and differentiated into dendritic cells under controlled conditions (Step 2). These cells are then engineered to express tissue-specific exosomal membrane proteins, facilitating targeted cargo delivery. Purified exosomes are loaded with therapeutic molecules such as miRNA or siRNA (Steps 3–4) and undergo rigorous quality control before being administered back to the patient (Step 5). This approach leverages autologous materials to enhance biocompatibility, minimize immunogenicity, and achieve precision targeting, representing a hybrid of natural and engineered therapeutic strategies. The figure was created using www.BioRender.com [157] (accessed on 6 January 2025).

Furthermore, Table 4 shows that autologous-derived exosomes, sourced from a patient’s own cells, offer the advantage of low immunogenicity, minimizing the risk of adverse immune responses and enabling personalized approaches for gene or drug delivery. These exosomes are directly obtained from specific tissues or biofluids of the patient, preserving the biological compatibility with the recipient. However, challenges related to scalability and cost-effectiveness remain significant barriers to their broader clinical adoption [82,148]. In contrast, self-derived exosomes are generated from hematopoietic stem cells (HSCs) mobilized from peripheral blood and engineered to express tissue-specific membrane proteins, offering enhanced targeting and scalability for broader therapeutic applications. This allows precise targeting of diseased cells and efficient delivery of therapeutic cargo, such as siRNA or miRNA, for disease modulation [82,146,149]. Unlike autologous exosomes, which are limited by their source tissue, self-derived exosomes are produced under controlled conditions, offering greater customization and scalability for therapeutic applications. As shown in Figure 3, therapeutic molecules can be loaded into self-derived exosomes during production, creating a highly personalized and efficient platform for delivering genetic materials to recipient cells. Rigorous quality control ensures their safety and efficacy prior to administration, making these vesicles a transformative tool for precision gene editing and targeted therapies [61,150,151].

**Table 4:** Clinical trials and therapeutic applications of exosome-based technologies.

Exosome Source	Therapeutic Application & Strategies	Clinical Trial Identifier & References
MSC-derived	Chemotherapy delivery (doxorubicin, paclitaxel) and tissue repair/anti-aging treatments through regenerative applications	NCT03608631, NCT05813379 [114,152]
Tumor-derived	Targeted siRNA delivery for KRAS-mutant cancers using precision RNA-based gene silencing Gene editing delivery (CRISPR-Cas9 systems) through genetic editing of oncogenes	[86,102]
Synthetic	IFN-y-enhanced immune therapy for solid tumors to deliver enhanced immunotherapy	[92,93]
Preconditioned	Hybrid vesicles for cancer therapy combining natural exosomes with synthetic elements LDLR mRNA delivery for familial hypercholesterolemia using biocompatible drug delivery systems	[91]
Artificial	Delivery of curcumin for colon tissue inflammation via biocompatible oral drug delivery	[42,100]
Plant-derived	Personalized gene or drug delivery for cancer, leveraging patient-specific biocompatibility to minimize immunogenicity	NCT05043181, NCT01294072 [65,66,97]
Milk-derived	Delivery of curcumin for colon tissue inflammation via biocompatible oral drug delivery	[96]
Autologous-derived	Personalized gene or drug delivery for cancer, leveraging patient-specific biocompatibility to minimize immunogenicity	[98,99]
Self-derived	Gene therapy platforms using peripheral blood-derived hematopoietic stem cells engineered to deliver siRNA or miRNA cargo	[100]
siRNA-Loaded	Gene silencing therapies targeting oncogenic mutations like KRAS, enhancing therapeutic precision in oncology	NCT05043181, NCT01294072 [132,133]

As highlighted in Table 4, the diverse therapeutic applications of exosomes emphasize their adaptability in addressing a variety of medical challenges. Whether in chemotherapy, gene therapy, immune modulation, or regenerative medicine, these vesicles offer a transformative platform for advancing precision medicine. Their ability to combine natural properties with synthetic modifications further enhances their therapeutic potential, providing innovative solutions to unmet clinical needs [132,153,154,155,156].

Building on the diverse therapeutic applications outlined in Table 4, small interfering RNAs (siRNA)-loaded exosomes stand out as a precision tool for targeted gene therapy. These vesicles leverage the inherent advantages of exosomes—biocompatibility, low immunogenicity, and efficient cargo delivery—to enhance the specificity and stability of siRNA therapies. Their ability to protect siRNAs from enzymatic degradation and minimize off-target effects positions siRNA-loaded exosomes as a transformative approach for treating gene-driven diseases, particularly in oncology.

### 8.2. siRNA-Loaded Exosomes: A Precision Therapeutic Tool

The use of siRNA-loaded exosomes represents a breakthrough in precision medicine, leveraging the unique properties of exosomes to deliver siRNAs directly to target cells [158]. This innovative approach minimizes off-target effects, enhances therapeutic specificity, and holds significant promise for treating gene-related diseases, particularly in oncology [159]. Exosomes serve as natural carriers for siRNAs due to their biocompatibility, low immunogenicity, and ability to traverse biological barriers, such as the blood–brain barrier [160]. By encapsulating siRNAs within their lipid bilayers, exosomes protect these fragile molecules from enzymatic degradation during systemic circulation, ensuring efficient delivery to target cells.

**Figure 4:**
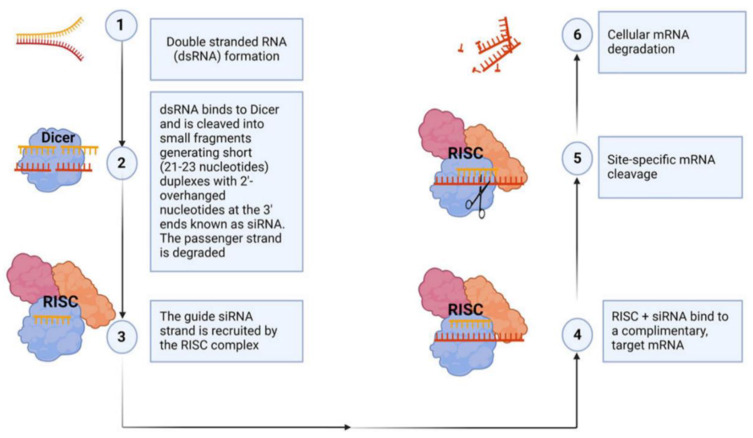
siRNA mechanism of action. This figure illustrates the evolutionarily conserved process of RNA interference for gene silencing. The mechanism begins with the recognition and cleavage of long double-stranded RNA (dsRNA) by Dicer, a ribonuclease, into short interfering RNA (siRNA) duplexes (steps 1–2). These siRNAs typically contain 20–24 base pairs with two-nucleotide overhangs and phosphorylated 5′ and hydroxylated 3′ ends. The siRNA duplex is then incorporated into the RNA-induced silencing complex (RISC), where the passenger strand is degraded by Argonaute 2, leaving the guide strand intact (step 3). The RISC complex, guided by the siRNA guide strand, binds to complementary sequences in the target mRNA (step 4), resulting in site-specific cleavage of the mRNA (step 5). This catalytic cleavage leads to the degradation of the mRNA (step 6), effectively suppressing gene expression. Synthetic siRNAs can be introduced into cells to target specific genes with complementary sequences, enabling functional validation and potential therapeutic applications. Figure 4 above was adapted from Ubanako et al., 2024 [143].

Once internalized, siRNA-loaded exosomes initiate gene silencing. As illustrated in Figure 4, siRNAs are incorporated into the RNA-induced silencing complex (RISC) within the target cell. The RISC–siRNA complex binds specifically to complementary mRNA sequences, facilitating mRNA cleavage to and subsequent gene silencing [46,161,162]. This highly targeted mechanism effectively silences disease-related genes through precise downregulation [46,161,162]. The inherent ability of exosomes to shield siRNA from nuclease-mediated degradation and evade immune responses enhances their safety and efficiency, making them superior to synthetic delivery systems. Additionally, exosomes can be engineered with surface ligands for targeted delivery, improving cell-specific uptake and therapeutic precision. Recently Kim et al. utilized next-generation RNAi therapeutics to demonstrate that exosomes, by leveraging their inherent strengths, can effectively deliver siRNA to target oncogenic drivers such as KRAS mutations, modulate angiogenesis through the silencing of Vascular Endothelial Growth Factor Receptor-2 (VEGFR-2), and counteract chemoresistance by disrupting metabolic pathways, thereby providing a personalized and efficient approach to cancer treatment [163].

Despite their advantages, the clinical translation of siRNA-loaded exosomes encounters significant hurdles. Achieving standardized and scalable production of exosomes remains technically complex, with variations in exosome composition and siRNA loading efficiency posing potential limitations to therapeutic efficacy [164]. Furthermore, refining targeting strategies to ensure precise delivery to diseased cells while sparing healthy tissues remains a key focus. Innovations such as stimuli-responsive delivery systems, which utilize external triggers (e.g., pH, temperature, or enzymes) to control the release of therapeutic cargo at specific sites, offer promising solutions for enhancing precision and efficiency [165]. Additionally, leveraging artificial intelligence (AI) in the design and optimization of delivery mechanisms could further advance the practical application of siRNA-loaded exosomes, paving the way for minimally invasive therapies across a range of diseases.

Table 5 presents a detailed overview of clinical trials and studies exploring the therapeutic potential of exosome-derived therapies, categorized by phase, disorder, origin/source, isolation method, outcome, and references. The trials encompass a wide range of disorders, including cancer, COVID-19, chronic inflammation, and regenerative conditions such as acne scars and macular holes. Exosomes derived from diverse sources, such as MSC, dendritic cells, and umbilical cord tissues, highlight their versatility in addressing various pathological conditions. The primary isolation method across studies is ultracentrifugation, emphasizing its foundational role in exosome purification, while novel techniques like ExoSCRT™, which separates 0.1–0.5% pure exosomes from stem cells to create a highly concentrated and effective product, signal significant advancements in scalable and efficient isolation approaches.

The outcomes reported in these studies emphasize the significant therapeutic potential of exosome-based therapies, including their ability to induce antitumor immunity, mitigate cytokine storms, restore immune function, and enhance visual and tissue repair outcomes. In oncology, trials utilizing dendritic cell-derived exosomes (DEX)—exosomes isolated from dendritic cells that carry tumor antigens to stimulate T-cell responses—have shown promise for immune modulation and personalized cancer treatment. For instance, DEX has been evaluated in NSCLC and autologous dendritic cell-derived exosomes have demonstrated efficacy in metastatic melanoma. These findings highlight the pivotal role of exosome-based strategies in advancing immune-based therapies and precision oncology [166,167]. Similarly, studies targeting COVID-19 with exosomes highlight their therapeutic potential, particularly in reducing inflammatory responses and enhancing recovery in severe cases. However, challenges related to scalability and production efficiency must be addressed before these therapies can be broadly applied in pandemic scenarios. The progression of these trials, from pilot studies to Phase 2a, illustrates the increasing clinical confidence in exosome-based therapies, while highlighting the challenges of scalability, standardization, and heterogeneity. Emerging technologies and proprietary approaches offer promising solutions for improving the efficiency and consistency of exosome isolation and therapeutic application. Collectively, these findings highlight the transformative potential of exosomes in precision medicine, particularly in oncology, regenerative medicine, and emerging infectious diseases.

**Table 5:** Expanded applications of exosome therapeutics.

Phase	Disorder	Origin/Source	Isolation Method	Outcome	Reference
Pilot Randomized Clinical Trial	Malignant middle cerebral artery infarct	Placenta MSC-derived Exosomes	Ultracentrifugation	Supportive, restorative treatment	[67]
Phase I	Colorectal Cancer	Ascites-derived exosomes (Aex) + GM-CSF	Ultracentrifugation	Induced antitumor immunity	[70]
Phase I	NSCLC	Dendritic cell-derived exosomes (DEX)	Ultracentrifugation	Well-tolerated, advanced NSCLC	[168]
Phase I	Metastatic melanoma	Autologous dendritic cell-derived exosomes	Ultracentrifugation	Feasibility, safety in metastatic melanoma	[169]
Phase 2a	Severe COVID-19	Adipose MSC-derived exosomes (haMSC-Exo)	Ultracentrifugation	Safe and well-tolerated	[114]
Pilot Trial	COVID-19 pneumonia	Umbilical Cord MSC-derived Exosomes	Ultracentrifugation	Safe and beneficial for COVID-19 pneumonia	[164]
Cohort Study	Severe COVID-19	Bone marrow MSC-derived Exosomes	Not Reported	Reduced cytokine storm, immune restoration	[152]
Randomized Clinical Trial	Chronic inflammation	Plasma enriched with extracellular vesicles derived from platelets,	Centrifugation	Successful treatment for chronic inflammation	[133]
Double-blind Phase I	Acne Scars	Adipose tissue stem cell-derived Exosomes	ExOSCRT™Technology	Improved acne scar healing	[61]
Early Phase I	Macular Holes	Umbilical cord MSC-derived Exosomes	Ultracentrifugation	Improved visual outcomes post-surgery	[62]

Furthermore, patient recruitment for exosome-based therapies depends on disease-specific eligibility criteria, such as prior treatment history, biomarker expression, and disease stage. Oncology trials, such as NSCLC studies (NCT01159288), enroll patients with advanced-stage disease (stage III/IV) who have undergone prior systemic therapy. In regenerative medicine, osteoarthritis trials (NCT04223622) focus on joint inflammation markers and radiographic progression, while macular hole studies (NCT03437759) select patients based on pre-existing surgical interventions and anatomical hole size. Similarly, in neurology, trials like Alzheimer’s disease (NCT04388982) include patients with mild to moderate cognitive impairment, ensuring targeted assessment of exosome-based interventions. The primary and secondary endpoints of exosome trials vary across indications. Phase I studies prioritize safety, tolerability, and dose-limiting toxicities (e.g., MSC-derived exosomes for severe COVID-19, NCT04493242). Phase II trials expand assessments to include tumor response rates (NCT03608631 for pancreatic cancer), cognitive function improvements (NCT04388982 for Alzheimer’s Disease), and wound healing outcomes (NCT01668849 for radiation-induced oral mucositis). The statistical methodologies applied in exosome-based trials depend on the study design and clinical objectives. Most trials remain in early phases, where descriptive statistics are primarily used to summarize safety and immune response data. In contrast, efficacy-focused studies, which are less common at this stage, employ Kaplan–Meier survival analysis, Cox proportional hazards models, and regression techniques to evaluate disease progression and treatment efficacy (e.g., pancreatic cancer trial, NCT03608631). Additionally, biomarker-driven ANOVA models are applied in studies such as Alzheimer’s disease (NCT04388982) to assess cognitive changes over time, while imaging-based assessments are employed in macular hole studies (NCT03437759).

Despite promising results, exosome-based therapies face challenges related to scalability, standardization, and regulatory pathways. Establishing GMP-compliant production methods and developing standardized exosome characterization techniques are critical for ensuring reproducibility and regulatory approval. Future research should focus on large-scale, randomized Phase III trials, integrating real-world evidence, biomarker-guided patient selection, and innovative statistical modeling to enhance therapeutic efficacy and clinical applicability.

## 9. Tumor-Derived Exosomes in Cancer Progression and Therapy

As molecular couriers, these nanoscale vesicles facilitate intercellular communication within the TME, delivering bioactive cargo that can reprogram recipient cells. This dual function allows exosomes to facilitate both tumor-promoting and immune-suppressive processes, as summarized in Table 1, showing their pivotal role in the complex interactions between cancer cells and their microenvironment.

### 9.1. Exosome-Mediated Communication and Remodeling of the Tumor Microenvironment

Exosomes, particularly TDEs, facilitate intercellular communication by transferring bioactive molecules through fusion with target cell membranes. These vesicles deliver specific proteins and miRNAs that alter recipient cell phenotypes, such as immune cells and fibroblasts, reshaping the TME [165]. TDEs promote tumor progression by inducing angiogenesis, modifying immune responses, and enhancing metastatic potential [170]. TDEs play a vital role in immune modulation by carrying immunological molecules such as PD-L1. Lyu et al. demonstrated that tumor-derived exosomal PD-L1 interacts with PD-1 receptors on T cells, suppressing their activation and inducing T cell exhaustion, which plays a significant role in resistance to immune CPIs in cancers such as NSCLC and melanoma [171,172]. Elevated exosomal PD-L1 levels are associated with systemic immune suppression and reduced CPI efficacy, making these exosomes promising biomarkers and therapeutic targets [141,171].

### 9.2. Angiogenesis, Vascular Integrity, and Tumor Metastasis

TDEs play pivotal roles in orchestrating cancer progression by influencing angiogenesis, vascular integrity, and metastasis. These nanoscale vesicles deliver bioactive cargo, including proteins, RNAs, and lipids, that actively remodel the TME to favor tumor survival and dissemination:

Angiogenesis and Vascular Remodeling: TDEs drive angiogenesis by delivering miRNAs such as miR-21-5p, which suppress inhibitors of VEGF signaling, thereby promoting vascular remodeling and ensuring an adequate nutrient supply to tumors [173]. Pro-angiogenic factors like VEGF-A and oncogenic drivers such as mutated KRAS within TDEs further stimulate vascular proliferation and remodeling, supporting tumor expansion and survival [174,175]. In addition to direct effects on endothelial cells, TDEs CAFs and macrophages, which secrete additional pro-angiogenic exosomes, amplifying the pro-vascular environment [176]. This network of interactions creates a dynamic and adaptable vasculature that facilitates tumor growth and progression.

Vascular Integrity Disruption: TDEs compromise vascular integrity by delivering miRNAs such as miR-105, miR-939, and miR-181c, which downregulate endothelial junctional proteins like VE-cadherin and ZO-1. This weakening of endothelial barriers enables tumor cells to infiltrate the circulation, facilitating intravasation and subsequent metastasis [45,177]. The disruption of vascular integrity also enhances vascular permeability, creating pathways for metastatic cells to spread.

Platelet Interactions and Pro-Coagulant States: TDEs also interact with platelets to promote thrombosis by transferring tissue factors, heat shock proteins (HSPs), and histones. These molecules contribute to the hypercoagulable state often observed in metastatic cancers, further facilitating tumor cell survival and dissemination within the bloodstream [178,179].

Extracellular Matrix Remodeling and Pre-Metastatic Niche Formation: The extracellular matrix (ECM) undergoes significant remodeling under the influence of TDEs. These vesicles stimulate the secretion of fibronectin and activate matrix metalloproteinases (e.g., MMP-1 and MMP-9), which degrade ECM components to create spaces for tumor cell migration and invasion [41,180]. Integrins carried by TDEs, such as α6β4 and αvβ5, guide tumor cells to specific organs, enabling the establishment of pre-metastatic niches [181]. ECM-enriched TDEs, particularly those carrying fibronectin, enhance adhesion, migration, and invasion through interactions with integrins and the activation of signaling pathways like Akt/mTOR. These processes not only support local tumor proliferation and survival but also facilitate metastatic colonization, highlighting the critical role of ECM remodeling in cancer progression [177,182,183].

Immune Cell Modulation and Immune Evasion: TDEs suppress anti-tumor immune responses by reprogramming macrophages into tumor-associated macrophages (TAMs), recruiting regulatory T cells (Tregs), and impairing the cytotoxic activity of natural killer (NK) cells and CD8+ T cells [184]. These immune-suppressive effects facilitate immune evasion and further support tumor growth and metastasis.

**Figure 5:**
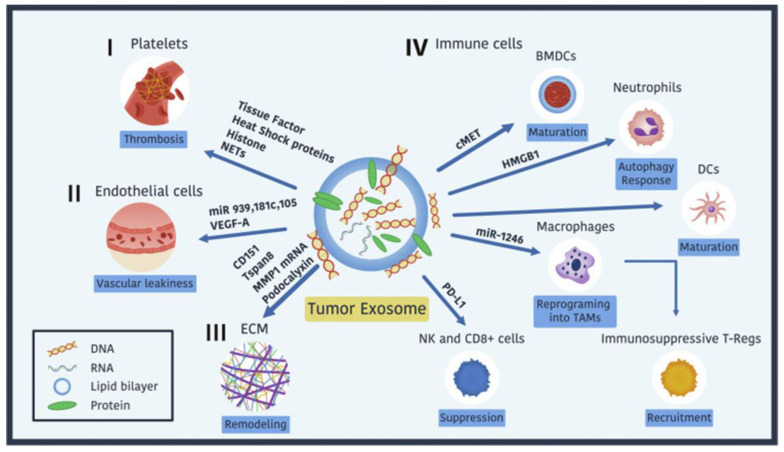
Tumor-Derived Exosomes in Cancer Progression. The figure illustrates how tumor-derived exosomes (TDEs), lipid bilayer-enclosed vesicles carrying diverse biomolecules (proteins, RNA, DNA, and glycans), contribute to cancer progression. These exosomes promote thrombosis through interactions with platelets, mediated by tissue factors, heat shock proteins, and histones. They induce vascular leakiness in endothelial cells via VEGF-A, podocalyxin, and exosomal miRNAs such as miR-939, miR-181c, and miR-105. TDEs also remodel the extracellular matrix (ECM) to facilitate tumor cell migration and invasion. Additionally, they modulate the immune system by suppressing NK and CD8+ T-cell activity through PD-L1, reprogramming macrophages into tumor-associated macrophages (TAMs) via miR-1246, recruiting immunosuppressive T-regulatory cells, and altering dendritic cell and neutrophil maturation and function. These mechanisms highlight the vital role of TDEs in reshaping the tumor microenvironment to support metastasis and immune evasion. Figure 5 was adapted from Wortzel et al., 2019 [45].

Figure 5 illustrates the multifaceted roles of TDEs in cancer progression. These lipid bilayer-enclosed vesicles carry diverse biomolecules (proteins, RNAs, and glycans) that interact with various components of the TME, including:

i. Platelets: Promote thrombosis via tissue factor, heat shock proteins (HSPs), histones, and neutrophil extracellular traps (NETs), facilitating tumor cell survival during circulation [155,156].
ii. Endothelial Cells: Enhance vascular leakiness by delivering VEGF-A and miRNAs (e.g., miR-939, miR-181c, miR-105) that downregulate junctional proteins like VE-cadherin and ZO-1 [157].
iii. ECM: Stimulate remodeling by inducing fibronectin secretion and activating MMPs (e.g., MMP-1 and MMP-9), creating pathways for tumor migration [158].
iv. Immune Cells: Suppress anti-tumor immune responses by reprogramming macrophages into TAMs, recruiting Tregs, and impairing NK and CD8+ T-cell cytotoxicity [158].

Recent studies have highlighted the potential of TDEs to modulate neutrophil activity via autophagic pathways. Exosomes carrying high-mobility group box 1 (HMGB1) and other danger-associated molecular patterns (DAMPs) activate neutrophils, promoting tumor-associated autophagic and inflammatory signaling cascades [185]. For instance, chronic stress has been shown to alter TDE secretion and cargo, leading to increased IL-1β production in neutrophils and facilitating lung metastasis in breast cancer [115].

This inflammatory niche, reinforced by TDEs, not only enhances immune evasion but also fosters conditions favorable for metastasis. Targeting TDE-induced neutrophil activation may provide new avenues for diminishing tumor progression while improving the efficacy of immunotherapies by disrupting the immune-privileged status of tumors.

### 9.3. Immunosuppression and Resistance Mechanisms

TDEs establish immunosuppressive conditions by transporting molecules like PD-L1, TGF-β, and miR-1246. These molecules reprogram immune cells into tumor-supportive phenotypes, such as TAMs, Tregs, and myeloid-derived suppressor cells (MDSCs), impairing the cytotoxic activity of NK and CD8+ T cells [106,107,186].

TDEs play a pivotal role in immune evasion and therapeutic resistance through multifaceted mechanisms. Glioblastoma-derived exosomes containing miR-1246 drive the expansion of immune-suppressive MDSCs, while leukemia-derived exosomes enriched with TGF-β hinder dendritic cell maturation, effectively suppressing the immune system’s ability to mount a response [106,107]. Beyond immune modulation, TDEs facilitate resistance to therapies by transferring key molecules, including drug-efflux pumps like P-glycoprotein, anti-apoptotic proteins such as Bcl-2 and survivin, and DNA repair proteins like ATM and Rad51, which collectively enhance tumor survival under therapeutic pressure [85,86,121]. TDEs establish immunosuppressive conditions by transporting molecules like PD-L1, TGF-β, and miR-1246, reprogramming immune cells into tumor-supportive phenotypes such as TAMs, Tregs, and MDSCs. They also contribute to therapeutic resistance by delivering key molecules like drug-efflux pumps and DNA repair proteins. Efforts to counteract exosome-mediated resistance include the use of exosome biogenesis inhibitors like GW4869, which suppress exosome biogenesis and limit the transfer of resistance-related molecules. GW4869 has shown efficacy in slowing prostate cancer progression by disrupting M2 macrophage differentiation induced by TDEs [122]. By targeting these mechanisms, therapeutic resistance and immune evasion can be mitigated, paving the way for more effective cancer treatments. In the hypoxic tumor TME, exosomes further exacerbate resistance and tumor progression by delivering HIF-1α and specific miRNAs that promote angiogenesis, metabolic adaptation, and therapeutic resilience, as demonstrated by Youssef et al. and colleagues [98,109,111,112,121].

### 9.4. Engineered Exosomes: Versatile Platforms for Therapeutic Innovation

Engineered exosomes represent a transformative frontier in precision oncology and beyond, highlighting great potential in addressing key challenges in modern medicine. Recent advancements have emphasized their ability to combat therapeutic resistance, enhance immune responses, and act as versatile carriers for precision therapies. As highlighted earlier, exosomes are being utilized as innovative vehicles for delivering siRNAs targeting oncogenic mutations, such as KRAS—a particularly challenging target in oncology. Additionally, exosomes carrying anti-PD-L1 proteins are driving advancements in immunotherapy by enhancing immune checkpoint blockade, thereby strengthening the immune system’s ability to combat tumors. These strategies also address chemoresistance, a significant obstacle to effective cancer treatment. By leveraging precise delivery mechanisms and tumor-specific targeting capabilities, exosomes reduce off-target effects, solidifying their role as promising tools in revolutionizing cancer care. Engineered exosomes hold immense promise in combating therapeutic resistance, boosting immune modulation, and enabling precision therapies, making them indispensable in the evolving landscape of oncology. Key oncologic applications of engineered exosomes include:

Gene Therapy and CRISPR-Cas9 Delivery: Engineered exosomes have been developed to deliver CRISPR-Cas9 systems targeting oncogenic mutations, such as KRAS G12C. This innovative approach allows precise genome editing, providing a promising avenue for addressing driver mutations in various cancers [39,110,113,118,157,187,188,189].

siRNA-Based Therapies: Exosomes loaded with siRNAs targeting oncogenes, like KRAS in colorectal cancer, effectively silence tumor-promoting genes. This method demonstrates the capability of exosomes to facilitate precision therapies by targeting molecular drivers of disease [40,163].

Immune Modulation: Engineered exosomes displaying tumor antigens can enhance T-cell and NK cell activity, boosting immune checkpoint blockade efficacy. By delivering anti-PD-L1 agents or amplifying immune-stimulatory signals, exosomes have redefined the landscape of cancer immunotherapy [185,189].

Combination Therapies: Exosomes carrying siRNAs targeting immunosuppressive molecules like TGF-β synergize with immune checkpoint inhibitors to amplify therapeutic effects, offering a multifaceted approach to overcoming resistance [177].

Drug Delivery and Chemoresistance Management: Exosomes improve the delivery of chemotherapeutics, enhancing efficacy and reducing systemic toxicity. They also counteract therapeutic resistance by transferring RNA-based agents, anti-apoptotic proteins (e.g., Bcl-2, survivin), and DNA repair proteins (e.g., ATM, Rad51) [85,86,118,121].

Sonodynamic Therapy (SDT): In a novel application, exosomes are being utilized as carriers for sonosensitizers in SDT. By leveraging ultrasound-activated sensitizers, these therapies generate reactive oxygen species that selectively induce cancer cell death, expanding treatment options for breast and other cancers [190].

Delivery of Noncoding RNAs: Exosomes delivering noncoding RNAs, such as microRNAs and long noncoding RNAs, can regulate gene expression in cancer cells. This approach has demonstrated success in inhibiting tumor progression and modulating key oncogenic pathways [108,191].

Cancer Vaccines: Exosome-based vaccines designed to present tumor antigens have shown significant promise in generating targeted immune responses. These vaccines are paving the way for personalized cancer immunotherapy strategies [166,167,192].

Hypoxic TME Adaptation: In hypoxic conditions, exosomes enriched with HIF-1α, and specific miRNAs promote angiogenesis, metabolic adaptation, and resistance. These findings indicate the adaptability of exosomes as mediators of tumor survival under adverse conditions [109,121].

### 9.5. Expanding Horizons: Exosomal Applications Beyond Oncology

Exosomes represent a cutting-edge advancement in therapeutic research, with applications spanning a wide range of disease areas. Their ability to deliver specific molecular cargo, such as RNA, proteins, and lipids, has made them invaluable in precision medicine. Below, we delve into the diverse roles of exosomes in various medical domains beyond oncology, highlighting their transformative potential as illustrated in Table 6.

Cardiovascular Medicine: In cardiovascular medicine, MSC-derived exosomes have shown remarkable potential in repairing vascular damage post-myocardial infarction (MI). These exosomes promote angiogenesis, reduce fibrosis, and modulate inflammation, contributing to enhanced cardiac function and tissue regeneration. Their multifaceted role in restoring cardiovascular health makes them an attractive candidate for regenerative therapies in heart disease.

Metabolic Disorders: Pancreatic beta cell-derived exosomes are at the forefront of metabolic disorder therapies. They have demonstrated the ability to restore insulin sensitivity, offering potential solutions for diabetes management. Additionally, plant-derived exosomes are being explored as scalable therapeutic carriers for metabolic regulation, providing an eco-friendly and efficient platform for addressing metabolic syndromes.

Bone Health: Exosomes derived from MSCs have been found to promote osteoblast differentiation and inhibit osteoclast activity, thus playing a vital role in bone regeneration. These exosomes can also be integrated into hydrogels to enhance their regenerative potential, paving the way for advanced treatments in bone health, particularly in conditions such as osteoporosis and fracture healing.

Autoimmune Diseases: Exosomes have emerged as key modulators of immune responses in autoimmune diseases such as lupus and rheumatoid arthritis. They deliver cytokine inhibitors and anti-inflammatory agents to affected tissues, providing targeted therapy with minimal systemic side effects. Additionally, exosomes have shown efficacy in sepsis-related diseases, further highlighting their potential in immune regulation and inflammatory disease management.

Neurological Disorders: Neuron-derived exosomes are being studied as biomarkers for neurodegenerative diseases like Alzheimer’s and Parkinson’s. These exosomes carry amyloid-beta (Aβ) and alpha-synuclein, enabling early diagnosis and disease monitoring. Furthermore, exosomes loaded with neuroprotective agents and siRNAs are being explored as therapeutic tools to modulate neuroinflammation, offering hope for conditions with limited treatment options [82,149,193].

Gastrointestinal Diseases: Gut microbiota-derived exosomes have demonstrated the ability to modulate inflammatory responses in CRC and inflammatory bowel disease (IBD). These exosomes act as therapeutic carriers for anti-inflammatory agents, providing targeted relief from gastrointestinal inflammation and associated complications.

Viral and Infectious Diseases: Exosomes carrying SARS-CoV-2 antigens are being developed as innovative platforms for next-generation vaccines. These exosomes mimic natural infection processes, thereby boosting immune responses and enhancing vaccine efficacy. Additionally, their anti-inflammatory effects hold promise in managing complications associated with COVID-19 therapy [119,194].

Liver Diseases: Regenerative exosomes are gaining traction in liver disease management, particularly in conditions such as cirrhosis. These exosomes promote liver repair by modulating immune responses and enhancing the regenerative capacity of hepatocytes. Their therapeutic potential is being explored as a viable option for chronic liver conditions with limited treatment alternatives.

Next-generation Vaccines: Engineered exosomes serve as platforms for next-generation vaccines. For instance, exosomes displaying decoy receptors for pro-inflammatory cytokines enhance efficacy in autoimmune and sepsis models [120].

**Table 6:** Advancements in exosome-based applications across therapeutic domains.

Disease Area	Exosome Applications	References
Cardiovascular Medicine	MSC-derived exosomes for vascular repair post-myocardial infarction (MI), angiogenesis promotion, and fibrosis reduction; modulation of inflammation for enhanced regeneration.	[63,124,195,196]
Metabolic Disorders	Pancreatic beta cell-derived exosomes restore insulin sensitivity; plant-derived exosomes as scalable therapeutic carriers for metabolic regulation.	[54,125,197,198]
Bone Health	MSC-derived exosomes promote osteoblast differentiation and inhibit osteoclast activity; exosome-integrated hydrogels enhance bone regeneration.	[116,125,126]
Autoimmune Diseases	Modulation of immune responses in lupus and rheumatoid arthritis; delivery of cytokine inhibitors; anti-inflammatory applications in autoimmune and sepsis-related diseases.	[39,54,197]
Neurological Disorders	Biomarkers for Alzheimer’s and Parkinson’s diseases (Aß, alpha-synuclein); therapeutic delivery of neuroprotective agents and siRNAs; modulation of neuroinflammation.	[39,63,197,199]
Gastrointestinal Diseases	Gut microbiota-derived exosomes modulate inflammatory responses in colorectal cancer (CRC); therapeutic anti-inflammatory agents for inflammatory bowel disease (IBD).	[197,198,200]
Viral and Infectious Diseases	Exosomes carrying SARS-CoV-2 antigens for vaccine development; anti-inflammatory effects in COVID-19 therapy.	[201,202,203]
Liver Diseases	Regenerative exosomes promote liver repair in cirrhosis; modulation of immune responses in liver-related conditions.	[197,204]
Vaccine Development	Engineered exosomes displaying viral antigens mimic infection processes, boosting immune responses and enhancing vaccine efficacy for infectious diseases.	[91,205,206]

In summary, exosomes offer a highly adaptable and effective platform for addressing diverse pathological challenges. Their ability to deliver therapeutic agents, modulate immune responses, and facilitate tissue regeneration highlights their transformative potential in clinical medicine.

### 9.6. Navigating Challenges in Exosome-Based Therapies: Opportunities for Transformation

Table 7 summarizes challenges and emerging solutions in exosome-based Therapies. One of the primary challenges in exosome-based therapies lies in the scalability of production, as generating clinical-grade exosomes in large quantities remains difficult. Emerging solutions, such as bioreactor-based systems, offer promise for scalable production, as highlighted in recent findings [123]. Another critical area is the lack of standardized isolation and characterization protocols, which has hindered consistency and reproducibility in research. The adoption of MISEV guidelines for standardization is paving the way for greater uniformity in the field [63].

Safety concerns, including the risk of off-target effects and immune activation, remain a significant hurdle. Advances in synthetic exosomes and precise surface engineering are addressing these issues by enhancing the specificity and reducing potential adverse reactions [153]. Additionally, low therapeutic cargo loading efficiency is a persistent obstacle. Innovative techniques, such as electroporation and sonication, are improving the ability to load exosomes with therapeutic agents effectively [124].

Another challenge pertains to the in vivo stability and biodistribution of exosomes, as they are often rapidly cleared and show inconsistent distribution in target tissues. Surface modifications aimed at enhancing targeting and stability have shown promise in overcoming these limitations [124]. Furthermore, the incomplete understanding of how exosomes interact with recipient cells hinders progress in therapeutic applications. Ongoing research into cellular and molecular pathways is gradually illuminating these mechanisms [125].

Regulatory challenges also pose significant barriers due to evolving compliance frameworks and ethical considerations. International collaborations and harmonization of regulatory standards are essential steps to ensure the safe and effective development of exosome-based therapies. Despite these challenges, exosomes are recognized as natural drug carriers with exceptional potential to transport biomolecules, positioning them as ideal therapeutic delivery systems. Efforts to explore both intrinsic and modified exosome properties are advancing this application [126].

In the realm of immunotherapy, exosomes show immense potential to modulate immune responses for cancer and autoimmune disease treatments. The development of immuno-engineered exosomes is opening new avenues for targeted therapies [116]. Similarly, in regenerative medicine, MSC-derived exosomes are proving to be powerful agents for tissue repair and regeneration. Expanding these applications to address more degenerative conditions is a priority for future research [114].

Finally, exosomes carry specific biomarkers that make them ideal for noninvasive disease diagnosis and monitoring. Advancements in biomarker discovery and validation are enhancing their utility in diagnostics (Biomed Central). Collectively, these emerging solutions are addressing key challenges and unlocking the transformative potential of exosomes in medicine, as reflected in the growing body of literature.

## 10. Unveiling the Role of Microbiome-Exosome Interactions in Cancer

The intricate interplay between the gut microbiota and exosome biology has emerged as a pivotal axis influencing cancer detection, progression, and therapy [152]. The gut microbiota, a complex ecosystem of trillions of microorganisms, profoundly impacts host immunity, metabolism, and cellular communication [209]. These functions are tightly linked to the modulation of circulating exosomes, which act as molecular couriers within the TME [210].

### 10.1. Microbiome Influence on Exosomal Cargo and Cancer Dynamics

Microbial populations modulate exosomal cargo composition, with significant implications for tumor progression and therapeutic outcomes. A study by Li et al. demonstrated how specific microbial communities shape exosomal content to enhance cancer stem cell (CSC) support, promote tumor progression, and reprogram the TME to favor therapeutic resistance [168].

Microbial dysbiosis—disruptions in microbial diversity—has been shown to impair exosome functionality. Ocansey et al. found that dysbiosis in inflammatory conditions like IBD alters exosomal cargo, facilitating immune evasion and metastasis by suppressing anti-tumor immune responses and promoting cancer cell dissemination [169].

### 10.2. Microbial Metabolites and Exosome Interactions

Microbiota-derived substances, such as short-chain fatty acids (SCFAs), further illustrate the relationship between gut health and cancer defense [207,211,212]. SCFAs, such as butyrate, modulate exosomal cargo by inhibiting histone deacetylases, altering signaling pathways to suppress tumor growth and metastasis [211]. In contrast, Pathogenic microbes, such as *Fusobacterium nucleatum*, exploit exosomal pathways to enrich exosomes with oncogenic miRNAs, including miR-1246, miR-92b-3p, and miR-27a-3p, facilitating Epithelial–Mesenchymal Transition (EMT) and tumor progression [208]. *Fusobacterium nucleatum*, exploits exosomal pathways by inducing CRC cells to release exosomes enriched with oncogenic microRNAs, including miR-1246, miR-92b-3p, and miR-27a-3p [208]. These exosomes facilitate EMT and promote tumor progression by transferring oncogenic signals to recipient cells [213].

### 10.3. Therapeutic and Diagnostic Opportunities

Modulating exosomal content through microbiome-targeted strategies offers promising avenues for improving cancer treatment outcomes [109]. Probiotic and prebiotic interventions, such as the use of *Lactobacillus rhamnosus* and *Bifidobacterium longum*, have shown potential to reshape exosomal cargo, enhancing anti-inflammatory and tumor-suppressive signals [214,215].

Microbial populations influence exosomal profiles, offering dynamic insights into disease progression and the underlying mechanisms of host–microbe interactions. For example, distinct exosomal profiles in CRC and breast cancer reveal cancer-type-specific mechanisms that could revolutionize liquid biopsy approaches for early detection, patient stratification, and real-time monitoring of therapeutic responses [216,217].

Microbiome–exosome interactions also open opportunities for precision oncology. By modulating the gut microbiota, researchers can enhance the efficacy of immune CPIs, reduce inflammation-driven tumor progression, and improve patient stratification for targeted therapies [218,219]. AI-driven models are decoding complex microbiome–exosome interactions, uncovering microbial influences on exosomal cargo and their role in therapeutic resistance, and paving the way for innovative cancer therapies [168,210,215].

### 10.4. Expanding Beyond the Gut Microbiome

While the gut microbiota plays a significant role, non-gut microbiomes, such as the oral and skin microbiota, also impact exosome-mediated cancer pathways [220]. These microbial communities influence systemic immune responses and exosomal cargo, expanding the scope of microbiome research in oncology. Environmental and lifestyle factors, such as diet, antibiotic usage, and toxin exposure, further shape the microbiome and, consequently, exosome-mediated cancer dynamics [220,221]. Addressing these modifiable factors could unlock new avenues for therapeutic intervention.

### 10.5. Microbiome–Exosome Interactions: Bridging Precision Oncology

The intricate interplay between the gut microbiota and exosomes plays a pivotal role in shaping systemic immunity, tumor progression, and therapeutic responses. Host-derived exosomes can regulate microbiota composition through their cargo, such as miRNAs, while microbiota-derived exosomes or metabolites modulate host immune pathways, metabolic signaling, and inflammation. Beneficial microbial metabolites like butyrate enhance gut barrier integrity and suppress tumor-promoting pathways, whereas harmful microbes, such as *Fusobacterium nucleatum*, exploit exosomal pathways to facilitate cancer progression.

**Figure 6:**
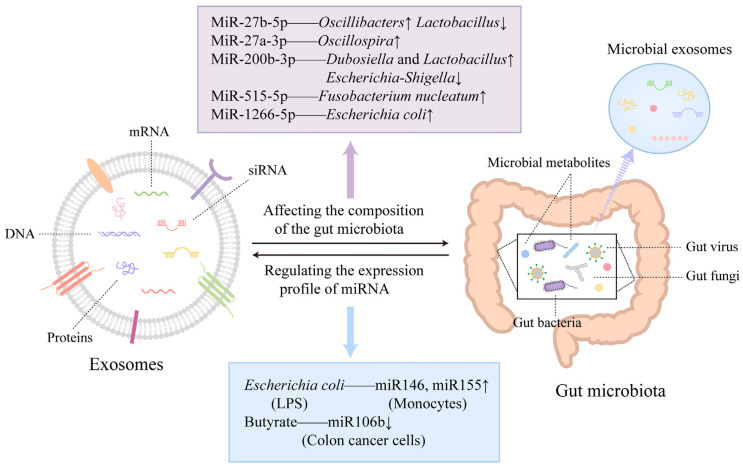
Microbiota–Exosome Interactions in Cancer Progression. This diagram illustrates the reciprocal relationship between gut microbiota and exosomes. Host-derived exosomes influence microbiota composition by delivering regulatory cargo, such as microRNAs (miRNAs), that modulate microbial diversity and activity. In turn, microbiota-derived exosomes and metabolites, such as short-chain fatty acids (SCFAs), impact host signaling pathways, immune responses, and metabolic processes. While beneficial microbes support anti-inflammatory and tumor-suppressive pathways, harmful microbes, such as Fusobacterium nucleatum, exploit exosomal pathways to promote epithelial–mesenchymal transition (EMT), metastasis, and immune evasion. These interactions represent a critical axis in understanding cancer biology and developing targeted therapies. Figure 6 was adapted from Cheng et al. (2024) [222].

Figure 6 illustrates these dynamic interactions, emphasizing the bidirectional communication between exosomes and gut microbiota. The figure highlights:

Microbial Metabolites and Exosome Modulation: Substances like butyrate regulate exosomal cargo, suppressing tumor-promoting pathways through miRNA modulation (e.g., miR-106b).

Oncogenic Interactions: Pathogenic microbes, such as Fusobacterium nucleatum and Escherichia coli, enrich exosomes with oncogenic miRNAs, including miR-27a-3p and miR-515-5p, promoting EMT and tumor progression.

Host-Exosome Influence on Microbiota: Host-derived exosomes, enriched with miRNAs like miR-146 and miR-155, regulate microbial populations and immune signaling pathways, influencing disease outcomes.

The interplay between the microbiome and exosomes is a promising frontier for developing innovative cancer therapies. This relationship leverages the ability of the microbiome to modulate exosomal cargo, influencing critical pathways in tumor progression and the TME. Targeting these interactions offers multiple therapeutic opportunities. For instance, probiotics and prebiotics, such as those promoting butyrate-producing bacteria, not only enhance gut health but also modulate exosomal cargo to carry anti-inflammatory and tumor-suppressive signals. Butyrate, a short-chain fatty acid produced by beneficial gut microbes, plays a pivotal role by altering exosomal cargo to suppress histone deacetylase activity, thereby regulating gene expression and inhibiting tumor-promoting pathways [214,215]. Son et al. and Gomes et al. demonstrated the key role of butyrate in regulating exosomal content and improving anti-tumor responses, paving the way for personalized cancer therapies that are both effective and minimally invasive [211,212].

Additionally, engineering microbiomes to influence exosomal profiles could further enhance treatment outcomes. Recent studies highlight that microbiota-targeted therapies can improve the efficacy of immune CPIs by modifying exosomal miRNA content to promote anti-tumor immunity Simpson et al. and Li et al. showed that probiotics such as *Lactobacillus rhamnosus* and *Bifidobacterium longum* reshape exosomal cargo, reducing inflammation and enhancing immune modulation. These strategies demonstrate the potential to integrate microbiome–exosome interactions into comprehensive therapeutic frameworks, advancing precision oncology and overcoming challenges like therapeutic resistance and immune evasion [211,218].

The integration of microbiome research with exosome-based diagnostics and therapeutics reveals new pathways for precision oncology [223]. Microbiome–exosome interactions influence systemic immunity, tumor progression, and therapeutic responses, highlighting their potential to expand exosome utility beyond tumor biomarkers [224].

## 11. Artificial Intelligence in Applications of Extracellular Vesicles and Exosomes

The integration of AI into EVs and exosome-based diagnostics and therapies is revolutionizing precision medicine, particularly in oncology. Exosomes, reflecting the physiological state of their originating cells, offer a wealth of biological insights. When combined with AI-driven data analytics, these vesicles enable enhanced diagnostic accuracy, optimized therapeutic strategies, and advanced patient-monitoring systems. This convergence marks a transformative era in cancer care and beyond [225,226].

### 11.1. AI-Driven Innovations in Exosome-Based Diagnostics and Therapies

AI technologies, including ML, DL, and natural language processing (NLP), excel in analyzing the complex molecular data embedded in EVs. These tools enable rapid, high-throughput analysis of EVs’ cargo, allowing for the automated detection of disease-specific markers with exceptional sensitivity and specificity [60,227]. For instance, AI simulations have revolutionized hybrid exosome design by enabling the precise selection and modification of surface ligands, enhancing cargo loading efficiency, and tailoring their structural properties. These advancements have refined drug-release dynamics to ensure controlled and sustained delivery and facilitated the creation of stimuli-responsive exosomes that release therapeutic agents with unmatched spatiotemporal precision, targeting specific cells or tissues in response to environmental triggers such as pH, temperature, or enzymatic activity [225,226]. Recent advancements reveal the potential of AI in diagnostics. Yin et al. employed ML to analyze serum-derived exosomes in CRC, using 4D-DIA proteomics to identify biomarkers like PF4 and AACT. Their AI-driven random forest model significantly outperformed conventional biomarkers, such as CEA and CA19-9, in sensitivity and specificity, demonstrating the utility of AI-enhanced exosome delivery systems for noninvasive CRC diagnostics [64].

Beyond diagnostics, AI redefines workflows for exosome analysis, enabling seamless transitions from sample processing to actionable clinical insights [204]. ML algorithms analyzing exosomal protein profiles achieved an AUROC score exceeding 0.91, indicating exceptional accuracy in distinguishing cancer-specific exosomes from non-cancerous ones. An area under the receiver operating characteristic curve (AUROC) score close to 1.0 reflects high sensitivity and specificity, demonstrating the effectiveness of ML in identifying subtle protein differences, crucial for early cancer detection and precise diagnostics [60]. Techniques such as label-free Surface-Enhanced Raman Scattering (SERS) reduce reliance on costly biochemical markers, optimizing resource use and identifying subtle disease indicators, including miRNAs and lipids, before clinical symptoms manifest [88,228]. In clinical settings, AI-driven tools have demonstrated significant utility. Aidoc’s radiology solutions, adopted by over 900 hospitals, and AEYE Health’s diabetic retinopathy screening system exemplify AI’s transformative potential. In oncology, ML algorithms have improved diagnostic accuracy by over 84% in cancers like colorectal, pancreatic, breast, and prostate cancer, enabling frequent liquid biopsies of exosomes for real-time monitoring of therapeutic responses [229,230].

By integrating reinforcement learning and variational autoencoders, GenAI accelerates preclinical screening, enhances early cancer identification during diagnostic and SOC screening, and streamlines the development of exosome-driven cancer therapies. Predictive modeling has improved delivery systems carrying siRNAs or CRISPR-Cas9, while tools like the Predictive Clinical Exosome Tool (PERCEPTION) integrate exosomal RNA data to forecast immunotherapy responses, enabling personalized treatment plans [231,232]. Generative Artificial Intelligence (GenAI) technologies, such as Generative Adversarial Networks (GANs), further enhance therapeutic design by modeling protein–ligand interactions. Ahmad et al. demonstrated the use of GenAI for tumor-targeting exosomal therapies, generating novel molecular structures and optimizing lead compounds. By integrating reinforcement learning and variational autoencoders, GenAI accelerates preclinical screening and streamlines the development of exosome-driven cancer therapies [233].

### 11.2. AI-Powered Evolution of EVs Biomarkers Beyond Oncology

AI is expanding the utility of EV-based diagnostics and therapies into non-oncology fields such as neurology, cardiology, and autoimmune disorders. As mentioned earlier, neuron-derived exosomes carrying amyloid β (Aβ) and tau proteins can predict Alzheimer’s disease progression years before clinical symptoms appear, enabling early interventions [226,234]. Similarly, cardiac exosomal miRNAs like miR-146a and miR-92b-5p are emerging as biomarkers for heart failure risks, aiding in preventive strategies [235,236,237,238]. Zhu et al. [40] demonstrated that AI-combined exosome profiling has revealed novel cardiac biomarkers capable of detecting dysfunction and tracking recovery post-intervention [40,237]. ML algorithms rapidly analyze exosomal data, uncovering biomarker signatures with unprecedented accuracy. For instance, convolutional neural networks (CNNs) distinguish neurodegenerative exosomal profiles from healthy controls with near-perfect sensitivity [239]. GenAI technologies also enable the engineering of synthetic exosomes tailored for specific diseases, transforming drug delivery and reducing development costs [240]. Real-time feedback systems powered by AI facilitate continuous monitoring of therapeutic responses via liquid biopsies of exosomes. This capability allows clinicians to dynamically adjust treatments, ensuring alignment with patient-specific outcomes.

The synergy between AI and EV-based diagnostics and therapies is redefining precision medicine. By streamlining workflows for disease detection, accelerating biomarker discovery, and delivering patient-centric care, AI enhances the clinical potential of exosomes. Applications extend beyond oncology to neurology, cardiology, and regenerative medicine, marking a new frontier in biomedical innovation [27,40,240].

### 11.3. AI-Driven Exosome Analysis in CRC: A Multi-Omics Model

AI is revolutionizing exosome research, particularly in CRC, by enabling the integration of complex multi-omics datasets, including those derived from the gut microbiota [197]. CRC represents a unique model disease in which AI-driven exosome analysis can unravel intricate molecular interactions between tumor biology, microbial dysbiosis, and host immune responses [195]. While CRC serves as an advanced example of AI-exosome applications, ongoing research is extending these methodologies to other cancers and diseases, emphasizing AI’s transformative potential in precision medicine [241].

A key advantage of AI in CRC diagnostics is its ability to integrate exosomal RNA sequencing (mRNA, long non-coding RNAs), cfDNA methylation profiling, and proteomic data to identify novel biomarkers for early detection and risk stratification. ML algorithms can analyze vast multi-analyte datasets to pinpoint discriminatory biomarker signatures with high sensitivity and specificity. For instance, AI-assisted feature selection can extract the most predictive exosomal RNA and protein markers, combining them into a multi-analyte signature with an AUC) of up to 0.99, demonstrating exceptional accuracy for minimally invasive CRC screening [197]. Moreover, AI allows researchers to correlate exosomal signatures with gut microbiota composition and function, providing insights into how microbial dysbiosis influences CRC pathogenesis and therapeutic response. These analytical approaches are being actively explored beyond CRC, with emerging studies applying AI-enhanced exosome diagnostics to lung, pancreatic, and breast cancers, as well as neurodegenerative and cardiovascular diseases [242].

Beyond diagnostics, AI is playing a pivotal role in predicting patient responses to CRC therapies by analyzing exosomal cargo and its correlation with treatment outcomes. Exosomal miRNAs and proteins can serve as indicators of chemoresistance and tumor aggressiveness, guiding personalized treatment strategies. ML models trained on exosomal miRNA profiles can predict resistance to chemotherapeutic agents such as 5-Fluorouracil (5-FU) or oxaliplatin, allowing for the early identification of non-responders and alternative therapy planning [243]. Additionally, AI can be applied to analyze exosome–microbiota interactions, as certain gut bacterial species influence miRNA expression and therapeutic resistance. This emerging field highlights the need for microbiome-aware AI models that consider the impact of host–microbe–exosome interactions on tumor progression and drug response [244].

AI is also driving exosome-based therapeutic innovations, particularly in targeted drug delivery for CRC. Computational models optimize exosome surface engineering, cargo loading, and receptor targeting to enhance precision therapy while minimizing off-target effects. A prime example is the AI-assisted design of exosomal carriers delivering siRNA or CRISPR-Cas9 therapies against KRAS mutations, which are prevalent in CRC [201,202].

AI algorithms can optimize exosome stability, internalization efficiency, and immune evasion properties, improving the efficacy of tumor-targeting exosomal vectors. Additionally, AI is facilitating the development of microbiota-modulating exosome therapies, where engineered exosomes deliver probiotics, bacterial-derived metabolites, or immune-modulatory molecules to the gut, reinforcing the host’s anti-tumor defenses. Similar exosome-based strategies are being actively explored for pancreatic and lung cancers, neurodegenerative diseases, and inflammatory disorders, broadening the clinical implications of AI-powered exosome engineering [245,246,247,248,249].

Beyond diagnostics and therapeutics, AI can facilitate a deeper understanding of the complex interactions within the TME in CRC by integrating spatial transcriptomics data with exosomal RNA profiling. Graph AI models can be used to analyze the spatial distribution of different cell types within the TME and to identify how exosome-mediated communication between these cells influences CRC progression [250,251]. This approach can reveal novel therapeutic targets and strategies for disrupting the pro-tumorigenic effects of the TME. By incorporating data on the gut microbiota and its influence on the TME, AI can provide a more comprehensive understanding of CRC pathogenesis and identify novel approaches for targeting the disease [252].

While CRC serves as a highly advanced example of AI-driven exosome analysis, the integration of AI, exosomal profiling, and microbiota data are rapidly expanding across multiple disease domains. With the ongoing refinement of AI models, multi-omics pipelines, and deep learning-based exosomal analysis, researchers are poised to redefine precision medicine through AI-exosome synergy, paving the way for early detection, personalized therapy, and microbiome-targeted interventions across a spectrum of human diseases [253].

## 12. Real-World Applications of Exosome-Based Diagnostics and Therapeutics

Exosome-based technologies are revolutionizing oncology, offering novel approaches for diagnostics, targeted drug delivery, and immune modulation. One of the most promising applications is their role in liquid biopsies, enabling noninvasive cancer detection by capturing tumor-specific markers with high sensitivity and specificity. For example, serum-derived exosomal PSA levels distinguish prostate cancer patients with remarkable accuracy [205], while ML models analyzing exosomal profiles in colorectal cancer outperform traditional biomarkers like CEA and CA19-9 [206]. Similarly, pancreatic cancer detection has been enhanced through GPC1-enriched exosomes, demonstrating exceptional sensitivity and specificity for early-stage diagnosis [91].

Beyond diagnostics, exosomes are now integral to therapeutic strategies, particularly in clinical trials. MSC-derived exosomes have shown immunomodulatory and regenerative potential in diseases such as graft-versus-host disease and chronic kidney disease [63,254]. Additionally, exosome-based RNA therapeutics, such as KRAS-targeted siRNA, have shown promise in silencing oncogenes in pancreatic cancer, as demonstrated in Phase I trials [39]. Advances in gene editing also leverage exosomal delivery of CRISPR-Cas9 components, enabling precise modifications of KRAS and TP53 while minimizing off-target effects [39,156].

In immunotherapy, exosomes are emerging as key players in enhancing immune responses. Tumor-derived exosomes carrying PD-L1 provide critical insights for developing novel checkpoint inhibitor therapies [141], while dendritic cell-derived exosomes presenting tumor antigens have been shown to enhance T-cell activation and improve immune responses in melanoma and NSCLC patients [253].

Exosomes also play a crucial role in metastasis management by facilitating tumor invasion and establishing pre-metastatic niches. Specific exosomal microRNAs, such as miR-1247-3p, activate CAFs, leading to cytokine secretion that promotes metastasis [96]. Moreover, exosomal integrins (e.g., αvβ5, α6β4) have been implicated in organ-specific metastasis in pancreatic and breast cancers [175], while miR-25-3p in colorectal cancer-derived exosomes remodels endothelial cells to create a pro-metastatic niche in the liver [199].

Together, these findings underscore the growing potential of exosomes in oncology, not only as diagnostic and prognostic tools but also as therapeutic vehicles capable of precise drug delivery and immune modulation.

## 13. Regulatory and Ethical Considerations

The integration of EVs-based technologies into precision medicine offers transformative potential but raises critical regulatory and ethical challenges [255]. The evolving frameworks for these diagnostics and therapeutics must address key issues such as classification—whether exosomes should be regulated as biological products, drugs, or medical devices—and quality control to ensure consistency and safety. Harmonized guidelines are essential to resolve ambiguities in their production and application, including ensuring traceability of exosome products and transparency in their development and use. Minimal Information for Studies of Extracellular Vesicles (MISEV) 2023 provides a refined framework for EVs research, emphasizing rigorous nomenclature, reproducibility, and transparent reporting. These guidelines standardize experimental approaches, enhancing the quality and comparability of EVs studies and paving the way for broader clinical and translational applications [256,257]. Adhering to GMP is particularly vital to mitigate risks and build trust among stakeholders. In the United States, exosome products are regulated as drugs and biological products under both the Public Health Service Act and the Federal Food, Drug, and Cosmetic Act [255].

While the therapeutic potential of exosomes spans diverse fields—including neurodegenerative diseases, cardiovascular medicine, and regenerative therapies—their clinical translation is hindered by challenges in scalability and standardization. Achieving universally accepted isolation and characterization protocols is essential to ensure consistency and reproducibility across applications, especially when targeting non-oncology indications [258,259,260]. There are currently no Food and Drug Administration (FDA)-approved exosome products and the FDA has already issued safety notices and consumer alerts regarding unproven exosome products, highlighting the need for proper regulatory compliance [255]. This classification requires premarket review and approval, reflecting the FDA’s commitment to ensuring safety and efficacy. In Japan, Pharmaceuticals and Medical Devices Agency (PhMDA) has taken a different approach, categorizing exosomes as biologics subject to the same regulatory requirements as vaccines and blood products [198,255]. The European Medicines Agency (EMA) distinguishes between EVs, including exosomes, containing functionally translated RNA, which are classified as advanced therapeutic medicinal products (ATMPs), and those directly purified from cells, which fall under biological specifications [255].

Beyond these foundational regulatory concerns, ethical considerations play a pivotal role in the responsible implementation of EVs-based therapies. Informed consent is critical, given the unique nature of EVs- and exosome-derived products, often sourced from patient biofluids or cells. Patients must fully understand how their materials are used, especially in contexts involving genetic modification or biobanking for future research. Transparency about the risks, benefits, and long-term implications of participating in trials or receiving exosome therapies is essential for ethical compliance [84].

Equitable access to these technologies represents another significant challenge. High production costs and reliance on advanced infrastructure may limit the availability of EVs therapies to resource-rich regions, widening existing disparities in global healthcare [255]. Strategies to address this include capacity building in underserved regions, equitable pricing models, and international collaborations to ensure broader accessibility [196,200]. Without proactive measures, the promise of exosome-based precision medicine could remain confined to affluent populations, further exacerbating healthcare inequities. Furthermore, the integration of AI into EVs research introduces additional ethical complexities. While AI greatly advances the analysis of EVs, particularly their exosomal cargo and biomarker identification, it poses a risk of perpetuating biases if the training datasets lack representation of diverse populations [261]. Transparency in AI algorithms, accountability for outcomes, and efforts to include underrepresented groups in data collection are essential to ensure equitable and accurate application of AI-driven exosome technologies [258,259,262].

Maintaining public trust in vesicle innovations is essential, as the unregulated use of exosome therapies in some sectors has highlighted the need for robust governance and public education to combat misinformation. Open communication about the scientific rigor, safety, and efficacy of these technologies, paired with mechanisms to monitor their ethical application, is crucial for building trust. Addressing these regulatory and ethical challenges requires a unified framework that prioritizes safety, transparency, and inclusivity, harmonizes global standards, fosters interdisciplinary collaboration, and ensures that exosome-based precision medicine achieves its transformative potential as an equitable healthcare innovation [84]. The MISEV2023 guidelines further reinforce the importance of rigor, transparency, and reproducibility in EVs research by emphasizing detailed reporting, quality control, and the proper management of co-isolated contaminants. These practices not only ensure reliable and reproducible studies but also uphold ethical standards, paving the way for the safe and effective clinical translation of EVs-based technologies and driving impactful advancements in research and innovation [203].

## 14. Interdisciplinary Collaboration: Shaping the Future of Exosome Research

The advancement of exosome research demands a concerted effort across diverse disciplines, including oncology, bioengineering, and data science. This interdisciplinary approach is essential to address the complexities of exosome biology and unlock their full potential as diagnostic and therapeutic tools in cancer care.

### 14.1. Multidisciplinary Roles in Exosome Research

Exosome research thrives on multidisciplinary collaboration, with each field contributing unique expertise to advance clinical applications.

Oncologists provide critical insights into disease mechanisms, tumor biology, and clinical needs, ensuring that exosome-based technologies align with real-world challenges in oncology. Their expertise is particularly valuable for identifying biomarkers, such as PD-L1-positive exosomes, which have been shown to influence immune checkpoint activity and predict patient response to therapies [141]. Additionally, oncologists guide translational research by correlating exosomal biomarker profiles with clinical outcomes, advancing personalized treatment strategies.

Bioengineers play a pivotal role in developing innovative platforms for exosome isolation, characterization, and therapeutic application. Advances in microfluidic systems have enabled high-throughput and efficient exosome isolation, addressing challenges of scalability and purity in clinical settings [54]. Similarly, bioengineers are driving the development of hybrid vesicles and stimuli-responsive exosome systems, which enhance targeted drug delivery and mitigate resistance mechanisms [145]. These technological breakthroughs make it possible to produce clinical-grade exosomes at scale, paving the way for their integration into precision medicine.

Data scientists contribute by applying AI and ML to analyze complex exosome datasets. AI-driven tools, such as the Predictive Clinical Exosome Tool (PERCEPTION), integrate exosomal RNA data to forecast responses to immunotherapies, thereby enabling precision oncology [232]. ML algorithms have also significantly improved biomarker discovery, as demonstrated by Yin et al. whose random forest model outperformed conventional methods in identifying colorectal cancer biomarkers from serum-derived exosomes. Such contributions not only enhance the diagnostic and therapeutic utility of exosomes but also facilitate real-time patient monitoring and adaptive treatment planning [64].

Collaborative efforts between these disciplines enable the integration of cutting-edge technologies, such as CRISPR-Cas9-loaded exosomes for gene editing [39], into therapeutic regimens. Moreover, interdisciplinary initiatives drive the standardization of protocols and the establishment of shared databases, promoting reproducibility and global adoption of exosome-based innovations.

### 14.2. Real-World Collaborative Case Studies

The integration of academic and industrial expertise has driven significant advancements in exosome research, addressing challenges in scalability, therapeutic translation, and regulatory compliance. Below are examples of successful interdisciplinary collaborations:

Therapeutic Innovations in Neurodegenerative Diseases: The partnership between Celltex Therapeutics and Texas A&M Institute for Regenerative Medicine explored the potential of MSC-derived exosomes in treating Alzheimer’s disease. Academic researchers at Texas A&M focused on isolating and characterizing exosomes with neuroinflammatory-modulating properties, while Celltex advanced these findings through preclinical studies (NCT04855955). This collaboration exemplifies how academic expertise in fundamental research complements the industry’s focus on clinical translation [263,264].

Advancements in Rare Disease Therapeutics: EXO Biologics collaborated with academic researchers to develop EXOB-001, an exosome-based therapy for Bronchopulmonary Dysplasia (BPD) in preterm newborns. This partnership enabled one of the first EMA-approved clinical trials for MSC-derived exosomes (NCT06279741). It highlights the importance of interdisciplinary efforts in navigating regulatory pathways and addressing rare disease challenges.

Scaling Exosome Production for Clinical Use: The collaboration between Cytiva and RoosterBio addressed the bottleneck of scalable exosome production. Cytiva’s bioprocessing technologies combined with RoosterBio’s MSC platforms resulted in optimized production pipelines for clinical-grade exosomes. This innovation is a significant step towards making exosome-based therapies more accessible for large-scale clinical applications.

By combining the clinical acumen of oncologists, the technological expertise of bioengineers, and the analytical capabilities of data scientists, the field of exosome research is poised to transform precision oncology. This synergy accelerates the translation of laboratory findings into clinical practice, ultimately improving patient outcomes and shaping the future of cancer care. Together, these multidisciplinary contributions enable exosome research to address real-world challenges, advancing the field toward transformative diagnostics and therapeutics

## 15. Future Research Directions

The field of EVs, particularly exosome research, has witnessed significant advancements, yet numerous questions and challenges remain that warrant further exploration. Emerging research opportunities span foundational biological insights, translational applications, and regulatory frameworks.

Exosome Biogenesis and Molecular Composition: Investigating the specific molecular pathways involved in exosome biogenesis and cargo selection could reveal targeted strategies for therapeutic engineering. For instance, elucidating the role of ESCRT-independent pathways and lipid-driven mechanisms may enable precise cargo manipulation [14,18]. Understanding the factors influencing exosome heterogeneity, particularly in cancer-derived vesicles, remains a key challenge for developing targeted applications [22,24].

Advances in Isolation Technologies: Emerging isolation technologies, such as microfluidic platforms and TFF, hold promise for addressing scalability and reproducibility challenges. Future research should refine these techniques to ensure purity and yield while enabling clinical-grade applications [52,55]. Hybrid approaches combining traditional methods with ML could enhance the specificity of biomarker discovery [53,60].

Liquid Biopsy Applications: Expanding the clinical utility of exosome-based liquid biopsies necessitates addressing the challenges of biomarker validation and reproducibility. Collaborative consortia could standardize protocols and datasets to accelerate clinical adoption [85,86]. Future efforts should focus on identifying universal biomarkers for early detection, enabling broader application across diverse cancer types. Notably, proteins such as Clathrin Heavy Chain (CLTC), Ezrin (EZR), Talin-1 (TLN1), Adenylyl Cyclase-Associated Protein 1 (CAP1), and Moesin (MSN) have been identified as highly abundant universal exosomal biomarkers with significant diagnostic potential [47,60,89].

Therapeutic Applications: Advances in engineering exosomes for targeted delivery of therapeutic agents, such as CRISPR-Cas9 or siRNAs, are promising. However, more research is needed to improve loading efficiency, targeting precision, and scalability [44,118]. Exploration of hybrid exosomes that combine natural and synthetic properties may open new avenues for addressing therapeutic resistance and delivering personalized therapies [145,148].

Immune Modulation and Resistance Mechanisms: Exosome-mediated immune modulation and resistance to immune CPIs remain underexplored. Investigating strategies to reprogram TDEs or inhibit their immune-suppressive effects could yield novel therapeutic targets [141,171]. Understanding exosome interactions with immune cells in the TME will be crucial for developing immune-modulatory therapies [181,186].

Exosomes in Vaccine Development: Engineered exosomes presenting tumor antigens have demonstrated significant promise in generating targeted immune activation, paving the way for personalized cancer immunotherapy strategies [166,167]. Early-phase clinical trials with dendritic cell-derived exosomes have shown robust anti-tumor T-cell responses in melanoma and NSCLC patients, mimicking natural antigen presentation and achieving prolonged immune effects [166,167]. Advances in scalable production, such as tangential flow filtration and immunoaffinity capture, enhance their clinical feasibility [49,52]. Additionally, plant-derived exosomes provide a cost-effective, biocompatible alternative for vaccine delivery, further broadening their potential [61,62]. Future efforts should focus on standardizing exosome engineering and optimizing antigen loading to maximize their therapeutic impact in cancer and infectious diseases.

Microbiome–Exosome Interactions: While initial studies have established the influence of the gut microbiome on exosomal cargo, the bidirectional interactions between microbiota and exosomes in modulating systemic diseases remain underexplored [169,210]. Investigating microbiota-derived exosomes as biomarkers or therapeutic agents could provide innovative strategies for managing cancer, metabolic disorders, and inflammatory diseases [211,212].

Integration of Artificial Intelligence: AI has demonstrated potential in exosome biomarker discovery, diagnostics, and therapy optimization. Future research could focus on training algorithms with diverse, globally representative datasets to avoid biases and enhance generalizability [60,233]. Leveraging AI to model exosomal interactions within the TME or simulate therapeutic delivery could accelerate the clinical translation of engineered exosomes [204,225].

Long-Term Safety and Efficacy: The safety and long-term effects of exosome-based therapeutics, especially in gene editing and immune modulation, require comprehensive investigation. Preclinical and clinical studies should focus on understanding potential immunogenicity, biodistribution, and off-target effects [142,163].

Regulatory and Ethical Frameworks: The integration of EVs into clinical practice necessitates harmonized global regulatory standards. Research into ethical implications, particularly regarding patient-derived exosomal material and genetic modifications, will be critical for public trust and widespread implementation [255,257].

By addressing these questions, researchers can unlock the full potential of exosomes as diagnostic and therapeutic tools. These future directions aim to bridge the gap between innovation and implementation, advancing the field toward transformative clinical impact.

## 16. Conclusions

Exosomes have emerged as pivotal components in the rapidly advancing field of precision oncology. Their versatility as noninvasive biomarkers, therapeutic delivery vehicles, and modulators of immune responses positions them at the forefront of innovation in cancer care. These nanoscale vesicles bridge the gap between fundamental biological research and clinical applications, addressing critical challenges such as tumor heterogeneity, therapeutic resistance, and immune evasion.

Key advancements in exosome engineering, scalable manufacturing, and AI-driven analytics are accelerating the integration of exosome-based technologies into clinical practice. Liquid biopsies utilizing exosomal cargo have already demonstrated significant potential in early cancer detection and real-time therapeutic monitoring, offering a superior alternative to traditional methods. Similarly, exosome-based therapeutics, including gene editing and immunomodulation strategies, continue to show promise in both preclinical and clinical studies, underscoring their transformative potential.

Despite these advances, challenges persist. The heterogeneity of exosome populations, the need for standardized isolation and characterization protocols, and regulatory complexities remain barriers to widespread adoption. Addressing these issues will require interdisciplinary collaboration among researchers, clinicians, and regulatory bodies to establish universal guidelines and scalable solutions.

Looking ahead, exosomes hold the promise of revolutionizing cancer care by enabling personalized, minimally invasive therapies and enhancing our understanding of tumor biology. By overcoming current limitations and fostering global collaboration, exosome-based technologies can redefine the landscape of precision oncology, ultimately improving patient outcomes worldwide.

## Figures and Tables

**Table 1:** Mechanisms of exosomal immune modulations.

Mechanism	Exosomal Role	Example
T-Cell Activation	Presents tumor antigens via MHC molecules to stimulate T-cells.	DC-derived exosomes presenting antigens.
NK Cell Activation	Delivers activating ligands (e.g., NKG2D) and cytotoxic molecules.	NK cell-derived exosomes enhancing cytotoxic responses.
Counteracting Tregs	Reduces immunosuppression by decreasing Treg activity.	MSC-derived exosomes reprogramming the TME.
Checkpoint Synergy	Enhances CPIs by delivering modulatory signals directly to tumors.	Anti-PD-L1 exosomes in combination therapies.

**Table 2:** Exosome-based biomarkers in liquid biopsy: diagnostic and therapeutic applications.

Exosome Source	Biomarkers in Liquid Biopsy	Applications	References
CD63-positive exosomes	miRNA-21, HER2	Breast cancer monitoring and treatment resistance	[106,107,108]
CD81-positive exosomes	EGFRVIII, EGFR T790M	Lung cancer resistance tracking	[98,109,110]
Annexin V-positive exosomes	PSA, PSMA	Prostate cancer detection and aggressiveness monitoring	[111,112]
PD-LI and EGFR-positive exosomes	PD-LI, EGFR	Immune checkpoint activity monitoring and resistance tracking	[113,114]
GPC-1-positive exosomes	Biomarkers for pancreatic cancer	Pancreatic cancer detection	[115,116]
Exosomal circRNAs	Emerging biomarkers for chemoresistance	Chemoresistance tracking	[117,118]
Synthetic exosomes	Tumor DNA alterations	Cancer subtype stratification	[119,120]
Microbial-derived exosomes	miRNA modulation (e.g., miR-1247-3p)	Tumor microenvironment and metastasis tracking	[121,122]
Plasma exosomes	miR-15a-5p, miR-141, miR-210, miR-92a, miR-155	Endometrial cancer early detection, prostate cancer monitoring, pancreatic cancer early detection, colorectal cancer diagnosis, breast cancer prognosis	[106,123,124,125,126]

**Table 7:** Challenges and emerging solutions in exosome-based therapies.

Challenge/Opportunity	Details	Emerging Solutions	References
Scalability	Difficulty in large-scale production of clinical-grade exosomes.	Bioreactor-based systems for scalable production.	[166,167]
Standardization	Lack of uniform isolation and characterization protocols.	Adoption of MISEV guidelines for standardization.	[166,167]
Safety	Risk of off-target effects and immune activation.	Synthetic exosomes and precise surface engineering.	[141,172]
Loading Efficiency	Low efficiency of therapeutic cargo loading.	Advances in electroporation and sonication techniques.	[207,208]
In Vivo Stability and Biodistribution	Rapid clearance and inconsistent distribution of exosomes in vivo.	Surface modifications to enhance targeting and stability.	[163,208]
Mechanisms of Action	Incomplete understanding of how exosomes interact with recipient cells.	Continued research into cellular and molecular pathways.	[183,184]
Regulatory Issues	Evolving frameworks for compliance with GMP and ethical considerations.	International collaborations and regulatory harmonization.	[114,152]
Natural Drug Carriers	Exosomes’ ability to transport biomolecules positions them as ideal therapeutic delivery systems.	Exploring intrinsic and modified exosome properties.	[98,109]
Immunotherapy Applications	Potential in modulating immune responses for cancer and autoimmune disease treatments.	Developing immuno-engineered exosomes.	[180,181]
Regenerative Medicine	MSC-derived exosomes promote tissue repair and regeneration.	Expanding MSC applications to more degenerative conditions.	[168,169]
Diagnostic Biomarkers	Exosomes carry specific biomarkers for non-invasive disease diagnosis and monitoring.	Advancements in biomarker discovery and validation.	[163,166]

## Abbreviations

AACT: Alpha-Actin; AEYE: AI-Enhanced Eye Diagnostics; AI: Artificial Intelligence; AUC: Area Under the Curve; AUROC: Area Under Receiver Operating Characteristics; CAFs: Cancer-Associated Fibroblasts; circRNAs: Circular RNAs; CNF: Cellulose Nanofiber; CPI: Checkpoint Inhibitors; CRC: Colorectal Cancer; CRISPR: Clustered Regularly Interspaced Short Palindromic Repeats; CSC: Cancer Stem Cells; CT: Circulating Tumor; DHEAS: Dehydroepiandrosterone Sulfate; DIA: Data Independent Acquisition; DL: Deep Learning; DNA: Deoxyribonucleic Acid; ECM: Extracellular Matrix; EGFR: Epidermal Growth Factor Receptor; ELISA: Enzyme-Linked Immunosorbent Assay; EMT: Epithelial-Mesenchymal Transition; ESCRT: Endosomal Sorting Complex Required for Transport; EV: Extracellular Vesicles; EVS: Extracellular Vesicle Systems; FDA: Food and Drug Administration; FPLC: Fast Protein Liquid Chromatography; GMP: Good Manufacturing Practices; GPC: Glypican-1; HER2: Human Epidermal Growth Factor Receptor 2; HIF: Hypoxia-Inducible Factor; HSPs: Heat Shock Protein; IBD: Inflammatory Bowel Disease; ISEV: International Society for Extracellular Vesicles; IVFC: In Vivo Flow Cytometry; MISEV: Minimal Information for Studies of Extracellular Vesicles; Micro RNAs: miRNA; MISEV2023: Minimal Information for Studies of Extracellular Vesicles 2023; ML: Machine Learning; MMP1: Matrix Metalloproteinase-1; MMP9: Matrix Metalloproteinase-9; MSC: Mesenchymal Stem Cells; MVBs: multivesicular bodies; NK: Natural Killer Cells; NLP: Natural Language Processing; NSCLC: Non-Small Cell Lung Cancer; PCR: Polymerase Chain Reaction; PD: Programmed Death; PERCEPTION: Predictive Clinical Exosome Tool; PMN: Pre-Metastatic Niche; RES: Reticuloendothelial System; RNA: Ribonucleic Acid; RRR: Results, Reliability, Relevance; SE: Size Exclusion; SEC: Size-Exclusion Chromatography; SERS: Surface-Enhanced Raman Scattering; TAMs: Tumor-Associated Macrophages; TFF: Tangential Flow Filtration; TGF: Transforming Growth Factor; TME: Tumor Microenvironment; TP53: Tumor Protein P53; Tregs: Regulatory T cells; VE: Vascular Endothelial; VEGF: Vascular Endothelial Growth Factor Receptor.
